# The Hidden Routes of DNA Photostability: Charge and Proton Transfer in Excited Cytosine–Guanine Tetramers

**DOI:** 10.1021/acs.jpclett.6c00376

**Published:** 2026-05-07

**Authors:** Juliana G. de Abrantes, Josene M. Toldo, Mario Barbatti, Marco Sacchi

**Affiliations:** † School of Chemistry and Chemical Engineering, 3660 University of Surrey, Guildford, GU2 7XH, United Kingdom; ‡ Lyon 1 Université, ENS de Lyon, CNRS, Laboratoire de Chimie, UMR 5182, 69342 Lyon Cedex 07, France; § Aix Marseille University, CNRS, ICR, 13284 Marseille, France; ∥ Institut Universitaire de France, 75231 Paris, France

## Abstract

DNA’s extraordinary resistance to UV-induced damage—essential to the survival of genetic material since prebiotic times — stems from its ability to rapidly and efficiently dissipate electronic excitation energy through damage-free relaxation channels. Multiple decay pathways, at different time scales, have been identified. Yet, the detailed interplay of these competing decay pathways has remained elusive. Using nonadiabatic surface-hopping dynamics at the TD-CAM-B3LYP level, we investigate the excited-state behavior of DNA tetramers composed of stacked guanine–cytosine (GC)_2_ dimers in alternating and nonalternating sequences in the gas phase. Following photoexcitation, both systems populate a G → C charge-transfer state, with interstrand proton transfer emerging as the dominant relaxation mechanism. Overall, the simulations reveal a complex network of coupled charge- and proton-transfer events, highlighting the diversity and subtlety of DNA’s excited-state dynamics. These findings provide a mechanistic picture of how stacked bases in DNA efficiently funnel excitation energy back to the ground state.

## Graphical abstract

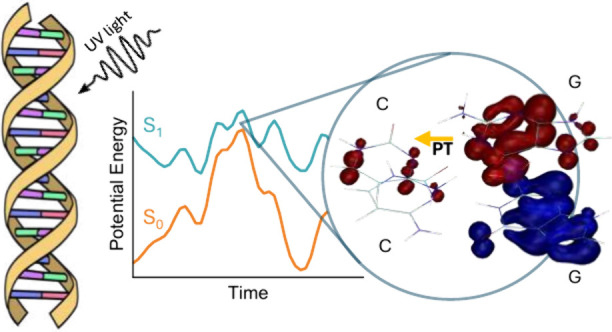

The interaction between radiation and DNA is fundamental to life on Earth, because it is involved in mutations, evolution, and even radiation therapy.^1–3^ Under UV irradiation, DNA is subject to potentially harmful reactions that can damage the nucleobases that constitute its macrostructure. Characterizing the photophysical and photochemical pathways in response to different radiation sources is, therefore, essential to understanding the mechanisms of DNA photodamage.

Still, DNA is known for its relative chemical photostability^4^ under UV irradiation, a feature that contributed to the successful relay of genetic information in the context of the origin of life.^5,6^ This is due to nonradiative relaxation pathways that can lead it back to the ground state.^7,8^ Its inherent stability is the reason why excited states in DNA have been the target of intensive study, not only for the indisputable biological relevance it entails in genetics, but also for being a test bed and benchmark for modeling reactions and relaxation mechanisms.^9^

A vast range of experimental and computational studies has investigated these processes, but they typically only provide a partial picture of the mechanisms that may occur. Due to computational limitations, most of the theoretical studies focus on single DNA nucleobases or nucleosides. In a nutshell, the main decay pathway described for nucleobase monomers is the ring puckering,^6,10–12^ leading to a conical intersection with the ground state. In dimers, other relaxation mechanisms emerge with the interbase interactions, always including charge-transfer (CT) states and often proton transfer (PT) between paired bases.^13–16^ In tetramers, where both stacking and hydrogen bonding interactions are present, there seems to be a competition of mechanisms^17^ including CT, single or multiple PTs, and also the formation of cyclobutane pyrimidine dimers (CPDs).^4,8,18^ Furthermore, tetramers of single-stranded DNA have also been computationally investigated, where it is shown that the excited state assumes a different character in the ultrafast regime before reaching a CT state between stacked bases, which leads to the ground state.^19^

However, the choice of system and method may introduce a bias in identifying which mechanisms are more significant in a scenario where many pathways compete, and there is still no definitive answer to which relaxation pathway following excitation dominates.^20^ For example, it is still under debate whether double tautomerisation occurs or not,^7^ if charge and proton transfer between base pairs are concerted or sequential,^6,17^ and whether single or multiple proton transfers prevail.^8^ Many studies highlight the need for a comprehensive dynamical analysis to clarify these open questions underlying the deactivation mechanisms.^17^ With this in mind, in this study we performed surface hopping dynamics using linear-response time-dependent density functional theory (TDDFT) with the CAM-B3LYP functional in tetramers composed of two-stacked base pairs of guanine and cytosine nucleobases in the gas phase. When constrained to a double-helix-like conformation, these tetramers provide the minimal model^17^ to account for hydrogen bonding and stacking interactions, both indispensable for describing the character of accessible electronic excited states. Given that stacking interactions impact orbital overlap, the deactivation mechanisms tend to be sequence-dependent,^21^ thus both alternating and nonalternating (GC)_2_ tetramers have been investigated (Figure 1).

**Figure 1:**
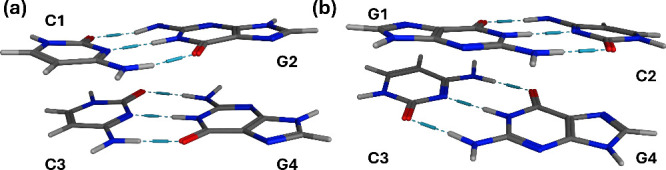
Structures of the (GC)_2_ tetramers: (a) nonalternating and (b) alternating sequences. The hydrogen bonds between Watson–Crick base pairs are shown in blue dashed lines.

Our results indicate that, once excited, the systems adopt a charge-transfer state from guanine to cytosine, with interstrand proton transfer being the dominant relaxation mechanism to S_0_ in both sequences. The CT state exhibits a purely interbase character in the nonalternating sequence and a mixed intra/interbase character in the alternating one, which might help further stabilize the system. However, the charges do not necessarily reside on the same residues involved in the proton transfer, and an interplay of charge separation, proton transfer, and charge rearrangement emerges.

The lowest three vertical excitations, oscillator strengths, and respective state character of alternating and nonalternating (GC)_2_ tetramers are shown in Table 1. The high charge transfer (CT) character number (GG → CC) shown in Table 1 (and further discussed in connection with Figure 5, presented later in this work), reflects the small overlap integrals between initial and final states upon absorption, explaining the low oscillator strengths. The alternating tetramer requires more than one orbital transition to describe the excitations and presents PR_NTO_ parameter of 0.25 (as further discussed); therefore, they are better classified as a charge resonance state. Depending on the method and geometry used, locally excited (LE) states might be found lying lower in energy than states with strong CT character.^6^ In a study using ADC(2), where four CGCG stacked bases along one strand are treated in the QM region, Plasser et al.^22^ found the first CT state to be S_17_. Therefore, the ordering of states must be considered *cum grano salis*, being that it is highly sensitive to the method and geometry chosen.

**Table 1:** Vertical Excitations, Oscillator Strengths, State Characterisation and Charge Transfer Character of the (GC)_2_ Tetramers Computed with TDDFT

	State	Energy (eV)	Oscillator Strength	Transition Character^a^	GG → CC CT Character
Nonalternating tetramer	S_1_	4.78	0.000	ππ* (0.98)	0.99
	S_2_	4.90	0.006	ππ* (0.92)	0.97
	S_3_	5.05	0.001	ππ* (0.95)	0.97
					
Alternating tetramer	S_1_	4.95	0.007	ππ* (0.62), ππ* (0.38)	0.93
	S_2_	4.96	0.013	ππ* (0.62), ππ* (0.38)	0.96
	S_3_	5.32	0.003	ππ* (0.47), ππ* (0.45)	0.23

a The respective percentage contribution of each state to the NTO absorption is shown in parentheses in the “Transition Character” column.

The first absorption peak with non-negligible oscillator strength is located at 4.90 eV (S_2_) for the nonalternating tetramer and 4.95 eV (S_1_) for the alternating one. These energies are in close agreement with experimental values, namely 4.86 eV for the former^8^ and 4.94 eV for the latter.^23^ The absorption spectra are shown in . The brightest state in both cases is the S_2_, and the main transitions correspond to a ππ* excitation (see the natural transition orbitals (NTOs) displayed in the ), where these transitions occur from a π orbital located on the guanine nucleobases to the π* on the cytosine nucleobases. For the alternating tetramer, the excitation corresponds to a linear combination of orbitals that, again, represent an absorption from the guanines to the cytosines of the tetramer. In isolated guanine and citosine, ππ* states are the lowest singlet excited states.^24^ Thus, in the tetramer, these nucleobase-centered ππ* excitations generate a band of low-energy states that dominates the bottom of the excited-state spectrum, as previously reported.^21,23^

For the nonadiabatic Fewest-Switches Surface Hopping (FSSH) dynamics, we considered the excitation energies and oscillator strengths of the Wigner-distributed geometries centered at 4.9 eV. This corresponds to the excitation to the brightest ππ* state. For the nonalternating tetramer, 14% of the trajectories were initiated in S_1_, 31% in S_2_, and 55% in S_3_. For the alternating tetramer, these fractions were 19%, 32%, and 49% for S_1_, S_2_, and S_3_, respectively. Therefore, the relaxation channels reported in this work are restricted to this excitation regime (i.e., excitation to the lowest bright state), being that if more bright ππ* states were initially populated, the observed dynamics might be affected.

Immediately after starting the dynamics propagation, a quick population transfer to S_1_ was observed. The red curve in Figure 2 reflects that this transfer occurs within less than 50 fs, as a consequence of relaxation of the higher excited states S_2_ and S_3_. A histogram of the times required for trajectories to reach S_0_ is shown in . On average, the trajectories decayed exponentially to the ground state with time constants of 64 fs for nonalternating and 141 fs for alternating base pairs.

**Figure 2:**
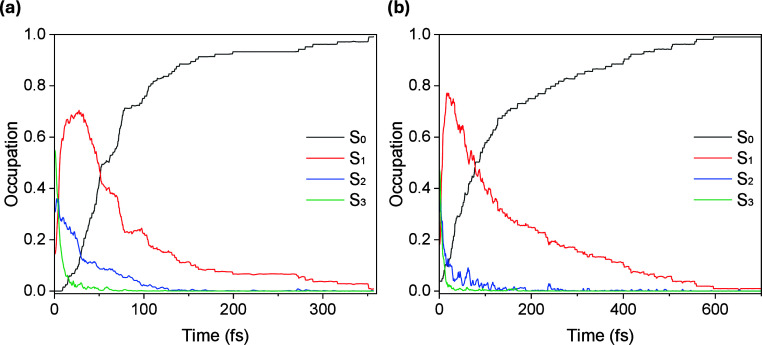
Time evolution of the adiabatic population (occupation) of (a) nonalternating and (b) alternating (GC)_2_ tetramers. The occupation of S_3_ state is represented in green, of S_2_ in blue, and of S_1_ in red, whereas the increasing population occupation of S_0_ is shown in black.

Figure 3 shows proton transfer times as a function of the time at which the corresponding trajectory populates S_0_ (which coincides with the final time of the trajectory, given that TDDFT, by construction, is inadequate to propagate the trajectory in the S_0_). We considered that a proton transfer event happened when the hydrogen atom reached X–*H* ≤ 1.1 Å on the opposite base, where X corresponds to any O or N atom on the Watson–Crick base pair. The many points for which PT is highly correlated with the S_1_ to S_0_ transfer times (final times), represented by points lying close to the diagonal in Figure 3, demonstrate the importance of PT as a mechanism of relaxation to S_0_. Points below the diagonal correspond to PT occurring before the decay to the ground state. Usually, points with the same S_1_ to S_0_ transfer time belong to the same trajectory, meaning that more than one PT has occurred along its propagation. For instance, in the plot regarding trajectories of the alternating tetramer (Figure 3b), the three points near 600 fs correspond to the situation where three PT events happened along that specific trajectory.

**Figure 3:**
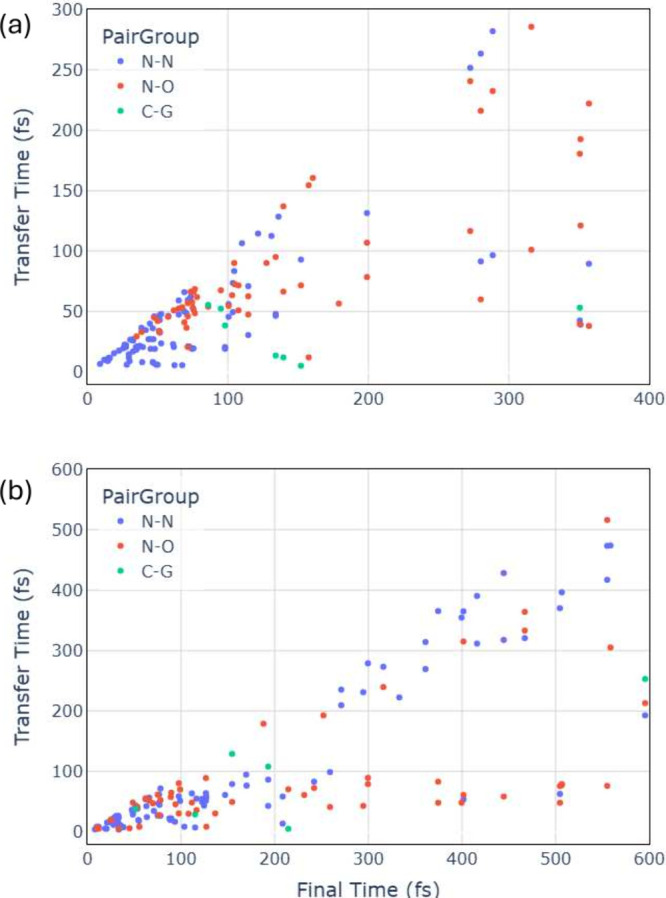
Proton transfer time vs trajectory final time in (a) nonalternating and (b) alternating (GC)_2_ tetramers. The final time of the trajectory corresponds to the moment the system relaxes to the ground state. The colors indicate the type of proton transfer that occurred: blue represents a hydrogen transfer on the central hydrogen bond, here labeled N–N; red is a transfer from guanine nitrogen to cytosine oxygen N–O; and green is a proton transfer from cytosine nitrogen to guanine oxygen, labeled as G–C.

From the correlation between PT times and the final times of the trajectories (Figure 3), it can be inferred that the primary mechanism responsible for relaxation to the ground state is the single proton transfer along the central hydrogen bond from guanine to cytosine (N–N transfer). This mechanism accounts for 82% of the cases in the alternating tetramer and 70% in the nonalternating one. The proton transfer from the amino group in guanine to the carbonyl oxygen in cytosine (N–O transfer) can also induce decay to the ground state (red points in Figure 3). It occurs in 24% of trajectories for nonalternating tetramers and 16% for alternating ones. The relevance of these two types of PT reproduces the trends reported by Francés-Monerris and co-workers,^8^ who also observed that the system may remain in the excited state after PT. In some cases, more than one type of PT occurs along the same trajectory, but in general, transfer rarely occurs from cytosine to guanine (dubbed C–G transfers in the figure). Indeed, the presence of tautomers arising from double proton transfer in C–G pairs has not been experimentally detected in stacked-base systems.^8^ However, this might also be attributed to the possibility of the protons returning to their nucleobase of origin once the system has relaxed back to the ground state.^25,26^ Finally, four trajectories finished without any permanent proton transfer in the (GC)_2_ nonalternating sequence, whereas nine such trajectories were observed in the alternating sequence.

The identification of single PT as a final relaxation mechanism is consistent with previous works,^8,17,27^ where the isolated nucleobase decay route of ring puckering is not favored due to a higher displacement along the mass-weighted coordinate,^28^ not due to a constraint artifact, since fixing the saturating hydrogens does not prevent the ring-puckering deactivation pathways.^29^ Likewise, these constraints should not affect which states are formed or the decay times, considering their distance to the π stacking sites that dominate interactions between bases. Additional benchmark calculations on a GC dimer along the proton-transfer coordinate show that the relative ordering of locally excited and charge-transfer states depends on both the electronic-structure method and the starting geometry (see ). In particular, TDDFT yields a stronger stabilization of CT character than CASPT2 ^26^ for paths initiated at the Franck–Condon minimum, whereas for geometries drawn from the Wigner distribution, the LE states remain competitive in the S_1_ region. These results indicate that the balance between LE and CT characters is method-dependent, but also sensitive to the part of configuration space sampled by the dynamics. Because the present tetramer has a much higher density of excited states and a larger hydrogen-bonding/stacking environment than the isolated GC dimer, these benchmark calculations do not provide a direct validation of the tetramer mechanism. They nevertheless show that the proton -transfer-driven decay reported here should be interpreted within the present TDDFT-based framework.

Regarding the charge-transfer character, within the ∼200 trajectories propagated in total, the electronic charges bounce between the fragments in a rather complex manner. A few representative movies illustrating the evolution of the geometries and electronic structures have been made available as . Nevertheless, three main patterns of proton/electron transfer could be identified. These patterns are shown in Figure 4, where solid arrows indicate the direction of proton transfer, while dashed arrows represent the direction of electron transfer at the moment when PT occurs.

**Figure 4:**
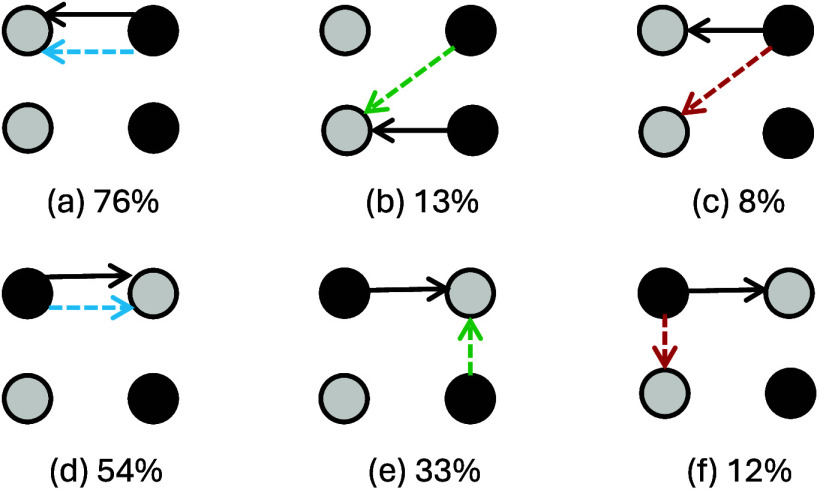
Scheme of main proton transfers, represented by solid arrows, and corresponding electron transfers, represented by dashed arrows, for nonalternating (a, b, and c) and alternating (d, e, and f) (GC)_2_ tetramers. Guanine is represented as a black circle and cytosine as a gray one. Under each scheme, a number denotes the percentage of the respective transfer pattern.

The most common case observed in both tetramers corresponds to PT occurring between the same fragments involved in the electron transfer (cases (a) and (d)), as previously described by Sobolewski, Domcke, and Hättig.^30^ In this case, the electronic charge transfer presents an interstrand character. This happens in 76.0% ± 8.9% of the proton transfer events in the nonalternating (GC)_2_ tetramer and 54.0% ± 10.1% of those in the alternating one. For the nonalternating tetramer, two other situations were identified. An electron transfer may occur from guanine to the *diagonal* cytosine in the π-stacked GC (G2 → C3 as labeled in Figure 1),^8^ giving rise to two distinct situations: either the cytosine, which is the proton acceptor, also serves as the electron acceptor (case (b), accounting for 12.5% ± 6.9% of PT events), or the guanine, which is the proton donor, simultaneously acts as the electron donor (figuring as the hole (h) in the NTO representation), accounting for 8.0% ± 5.7% of the cases and depicted as case (c) in Figure 4.

In the alternating tetramer, the electron transfer can, otherwise, assume an intrastrand character (cases (e) and (f)), meaning that the electron can be transferred to the π-stacked base on the same DNA strand. This directionality is not only in accordance with what has been proposed by Ko and Hammes-Schiffer^31^ (who showed that the intrastrand charge transfer state is lower in energy for the alternating sequence), but also supports the proton transfer induced by an intrastrand charge -transfer mechanism, proposed by Zhang et al.^32^ Again, there is either a cytosine that acts as an electron/proton acceptor (case (e), with 33.0% ± 9.5% of the cases), or a guanine that acts as an electron/proton donor (case (f), with 11.7% ± 6.5% of the cases). These mechanisms have been identified by Martínez-Fernández et al.^9,17^ and named PCET^1^ and PCET^2^, respectively. Notwithstanding, our results show longer decay time constants for the alternating tetramer, suggesting that bidirectional charge transfer might stabilize the system in the excited state. The remaining cases in which a C → G proton transfer occurred were too few to provide a significant proton/electron transfer pattern and, for this reason, are not shown in Figure 4.

Considering the directionality of charge transfer in Figure 4, although the proton displacement is triggered by the initial electronic charge separation, the proton and electron transfers do not necessarily occur on the same residues. Indeed, the overall picture for alternating and nonalternating tetramers is very similar: the dominant relaxation mechanism can be classified as excited-state hydrogen transfer (ESHT)^8^ or hydrogen atom transfer (HAT),^33^ in which an electron and a proton are transferred from the same base,^30^ resulting in a net hydrogen transfer.^4^ The second most common mechanism involves a proton and an electron moving toward the same acceptor cytosine. Finally, the third case corresponds to a proton and an electron departing from the same guanine base, thereby leaving a hole behind. These latter two mechanisms are more prominent in the alternating sequence, where electron transfer occurs along the same strand, likely due to a higher orbital overlap between the bases interacting through π-stacking. This process is classified as proton-coupled electron transfer (PCET),^13,32^ or electron–proton transfer.^33^ As pointed out in previous work, a complete neutralization of individual nucleobases is not expected, and different configurations can be achieved, including the presence of radicals and net ionic bases.^13^

Indeed, the excited states in the (GC)_2_ tetramers can display several distinct electronic characters, depending on how the electron (e) and hole (h) are distributed over the four nucleobases. As illustrated in Figure 5, we distinguish local excitation, excitonic resonance (exciton), charge transfer, charge resonance, and mixed LE+CR (excimer) patterns.^19,34^ To classify these states systematically and compactly, we use descriptors derived from the fragment-partitioned one-electron transition density matrix (1-TDM),^35^ treating each nucleobase as a single fragment. In practice, the classification is built around three complementary descriptors: (i) CT, the net electronic charge-transfer index, which quantifies electron–hole separation between fragments; (ii) PR, the electron/hole participation ratio, which measures delocalization over fragments; and (iii) POS, the average electron/hole position, which indicates which fragments predominantly host the e and h. In addition, we report PR_NTO_ as an auxiliary descriptor that can be useful to further discriminate between excitonic resonance (exciton) and charge resonance cases when the values of CT and PR yield similar combinations.^35^ Formal definitions of all descriptors are provided in the .

**Figure 5:**
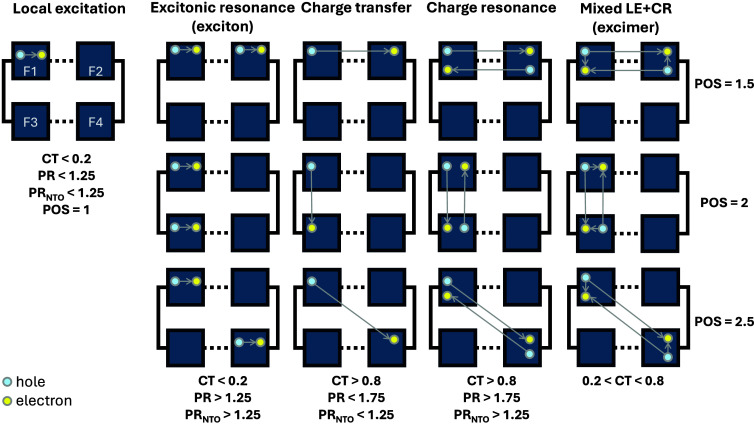
Diagram of possible excitations in the (GC)_2_ tetramers. The transitions can be classified according to CT and PR descriptors; the POS descriptor indicates which of the defined fragments are involved in the excitation; PR_NTO_ allows distinguishing excitonic and charge resonant states.

As depicted in Figure 5, when an electron and a hole are both positioned on the same fragment, a local excitation is defined. In this case, the average e/h delocalization should remain close to one, so we define a threshold of PR descriptor at 1.25, and for the charge transfer character (measured by the CT descriptor) at 0.2, as previously defined by Ibele et al.^19^ In cases where more than one local excitation occurs in different fragments, the delocalization descriptor increases (PR > 1.25) while keeping CT low. This excitation can be classified as excitonic resonance, following Plasser and Lischka’s nomenclature,^34^ or an exciton, according to Ibele et al.^19^

A charge-transfer state is defined when, upon excitation, the electron is transferred to a different fragment, which will result in a CT number larger than 0.8. If, however, both fragments present partial hole and partial electron character and, due to a charge exchange, there is no net charge transfer, a charge resonance state is defined, and the PR descriptor should get closer to 2.^34,36^ Note that the increase in the PR descriptor is an indirect indicator of charge resonance. The number of NTOs required to describe the excitation, PR_NTO_, is the appropriate descriptor to distinguish resonant states. Again, defining a threshold for PR_NTO_ > 1.25, reflecting a linear combination of configurations, correlates with PR > 1.75 values in the CT region (see ). The delocalization reflected by PR is therefore a consequence of the involvement of a higher number of orbitals in the excitation.^34^

Finally, an excimer is defined as a coherent superposition of Frenkel excitonic state and charge-transfer configurations (0.2 < CT < 0.8).^34^ It may or may not exhibit resonant character. In this case, due to a higher degree of charge separation, electron and hole can move more freely when compared to the exciton case,^37^ which preserves the concerted movement characteristic of a quasiparticle.

Figure 6 shows the distribution of the CT and PR descriptors sampled every 10 fs until the trajectory reached 50 fs, and every 50 fs after that. For visual guidance, the CT/PR plane is partitioned into five domains corresponding to the qualitative classes introduced in Figure 5 (local excitation, excitonic resonance (exciton), charge transfer, charge resonance, and mixed LE+CR (excimer)). We stress, however, that CT and PR provide only a two-dimensional projection of the excited-state character: in practice, the assignment also depends on which fragments host the electron and hole, captured by the POS descriptor (and, when needed, by PR_NTO_).

**Figure 6:**
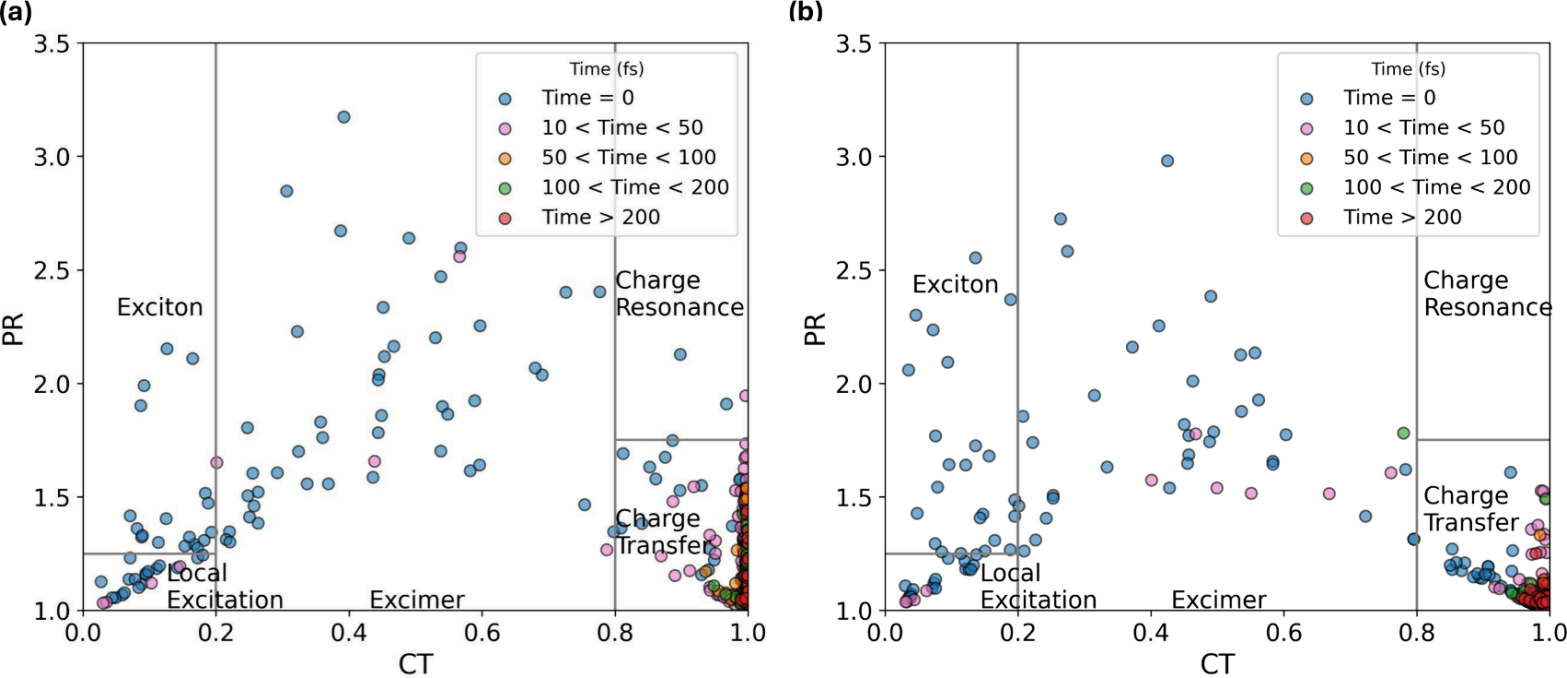
Sampling of CT and PR descriptors for the (a) nonalternating and (b) alternating (GC)_2_ tetramers. The legend indicates the time step at which these points were sampled. The plots are divided into five main regions, which allow classification of the excitation based on the descriptors.

The generation of initial conditions through a Wigner distribution creates an ensemble of structures slightly distorted from the equilibrium geometry. This distortion, combined with the high density of nearly degenerate excited states, may cause the initially accessed states to span different characters. As shown in Figure 6, the electronic populations are dominated by local excitation, excitonic resonance (exciton), and mixed LE+CR (excimer) character. This provides evidence of how sensitive the electronic structure is to the geometric conformation.

The distribution across an extensive PR window indicates that the average electron/hole position is delocalized over up to three fragments. Once the dynamics start, however, the systems quickly (*t* < 50 fs) assume a charge-transfer character, with the electron and hole positions well-defined over a guanine and a cytosine base, respectively. The rapid population of a CT state (CT > 0.8) had already been suggested by quantum dynamics calculations for the G–C base pair.^38^ As shown in Figure 6, after 50 fs, not only does the CT character reach its maximum, but the PR descriptor drops below 1.5, indicating that the electron and hole must now be localized on two different fragments, and generally do not exceed this value. The delocalized character of the excitation upon absorption versus a localized state at relaxation to the ground state had also been previously proposed by Martínez-Fernández and co-workers.^9^ A similar plot with points colored by PR_NTO_ values is shown in .

In general, the transitions with high CT character (CT > 0.8) do not require more than one NTO pair for their description (see ). In this sense, there are but a few cases of charge resonance, as defined by Plasser and Lischka^34^ in the nonalternating tetramer, meaning no exchange of charges between fragments. This condition is also reflected in the effective number of entangled states, the *Z*_HE_ descriptor. At higher times (*t* > 50 fs), *Z*_HE_ tends to 1, indicating that electron and hole wave functions become disentangled soon after the dynamics start developing in the excited state. show a plot of this descriptor sampled at specific times.

The analysis using the 1-TDM descriptors shows that, although the photoexcited systems initially populate a wide range of excited-state electronic characters, their evolution consistently leads to the formation of charge-transfer states before relaxation. This outcome is independent of the specific directionality of electron transfer preceding proton transfer, indicating that charge separation constitutes an important stage in the dynamics. In this sense, the mechanisms proposed in the literature are not mutually exclusive; instead, they represent different possible outcomes of a system that has been excited.

Overall, the trajectory ensemble shows a consistent early drift toward a charge-transfer character followed by relaxation events correlated with interstrand proton transfer, providing a unified dynamical picture across both alternating and nonalternating sequences. These results demonstrate how the nature of DNA photorelaxation is intrinsically diverse, and highlight the importance of considering both charge and proton dynamics when analyzing photoinduced processes in multichromophoric systems.

In summary, nonadiabatic dynamics simulations of alternating and nonalternating (GC)_2_ tetramers were carried out to identify the relaxation mechanisms that drive photoexcited DNA back to the ground state. The results reveal multiple competing pathways, uncovering a far more intricate and nuanced relaxation landscape than previously recognized. The systems rapidly form a charge-transfer state after photoexcitation, but the subsequent motion of the electron and hole can diverge significantly across trajectories. The many degrees of freedom and the complex coupling between stacked and paired bases allow the dynamics to unfold along several distinct routes, leading to a few possible relaxation mechanisms.

The primary decay pathway involves proton transfer between the nitrogen atoms of the central hydrogen bond between guanine and cytosine (N—N transfer), followed by a secondary N—O transfer within the same base pair. This interstrand proton motion is triggered by an initial charge separation, although the bases involved in each step are not always the same.

The trajectory-based FSSH picture emphasizes that relaxation in these tetramers is not confined to a single mechanistic channel: different trajectories follow different routes on a multidimensional excited-state surface whose topology is sequence-dependent.^17,31^ This reinforces that charge redistribution and interstrand proton transfer are intertwined processes during deactivation, which motivates analyzing both of them in photoinduced dynamics for multichromophoric systems.

Previous quantum-mechanical studies of G–C sequences have proposed that ultrafast population of G → C charge-transfer character can set the stage for interstrand proton-transfer channels, with the balance between pathways modulated by π-stacking, sequence, and structural fluctuations.^9,17^ Building on this picture, our surface-hopping dynamics provides a complementary, fully dynamical view of how these coupled charge and proton motions unfold in real time in (CG)_2_ tetramers. In particular, we find that the charge-transfer character and the proton-transfer events are correlated but not rigidly locked: the fragments carrying the dominant electron/hole density do not necessarily coincide with those involved in the proton-transfer step, and distinct deactivation routes emerge across the ensemble even within the same overall CT → PT pattern.

Finally, this work offers a framework for analyzing such complex, multipathway dynamics rather than proposing a universal reassignment of DNA photostability mechanisms. It should be kept in mind that this mechanistic picture emerges for this tetramer model within the current computational framework. In the future, combining surface-hopping with QM/MM would allow us to include solvent effects, which can promote energy dissipation and alter excited-state stability.^39^ Under the QM/MM framework, the explicit consideration of the DNA backbone in the MM region could modulate the conformational interplay of the nucleobase chromophores,^9^ which, in turn, might affect the charge distribution during the dynamics and enable outcomes such as cyclobutane pyrimidine dimer (CPD) formation to be assessed. It should be taken into consideration, however, that given that the formation of CPDs is a relatively rare event,^40^ running a statistically relevant number of trajectories would be required. We highlight that it is possible to use the fragment-based approach to follow the charge evolution also in the QM/MM framework, as long as the fragments to be considered are included in the QM region.

## Methods

Geometry optimizations of the alternating and nonalternating (GC)_2_ tetramers (Figure 1) were performed using DFT with the CAM-B3LYP functional,^40^ 6-31G* basis set^41^ and D4 dispersion corrections.^42^ CAM-B3LYP has been previously chosen for studying excited states in DNA, given its adequacy to describe charge-transfer systems,^8,19,38^ and 6–31G* basis set has also been used in recent studies,^21,38,43,44^ showing good performance while keeping the FSSH calculations to a reasonable cost. The carbon atoms connected to the backbone were saturated with hydrogen atoms, and their positions were frozen to simulate the rigidity of the backbone. The first three vertical excitations and the corresponding absorption spectra were computed with TDDFT at the same level described above.^31^ The coordinates of the tetramers’ ground state are reported in the .

Excited-state dynamics was performed with fewest-switches surface hopping (FSSH),^45^ including decoherence corrections (DC; α = 0.1 au).^46^ DC-FSSH is a nonadiabatic dynamics method that combines quantum equations to describe the electronic structure and classical equations to propagate the nuclei positions.^47^ The classical equations of motion were integrated with a 0.5 fs time step, while the quantum equations used a 0.025 fs time step, with interpolated electronic quantities. Time-derivative nonadiabatic couplings were estimated using the time-dependent Baeck-An approximation (TD-BA).^48^ The momentum adjustment after hopping was kept in the direction of the energy gradient difference, which prevents an excessive number of back hoppings.^49^ Since TDDFT fails to calculate regions near the S_1_/S_0_ crossing seam, only hoppings between excited states were evaluated with DC-FSSH. Trajectories were terminated when the energy gap between the excited and ground states was smaller than 0.2 eV. This termination time was assumed to correspond to the hopping time to S_0_. Throughout trajectory propagation, the saturating hydrogens were anchor-fixed by assigning them a mass of 999.00 u and setting their initial velocities to zero, thereby mechanically constraining their motion to mimic the effect of the DNA backbone.

A total of 100 trajectories were run for the alternating tetramer and 104 for the nonalternating one. Initial conditions were generated using a harmonic Wigner distribution around the equilibrium geometries. The absorption spectrum was computed using the nuclear ensemble approach.^50^ The initial state of each trajectory was selected to start from different excited states in proportion to their contribution to the absorption spectrum, centered at 4.9 eV for both tetramers. An energy window of ± 0.5 eV was used for the alternating tetramer, while a broader window of 0.9 eV was adopted for the nonalternating system in order to select a sufficient number of initial conditions for the subsequent dynamics.

DC-FSSH and spectra were performed with the Newton-X NS-V3 package.^51^ Statistical analysis of the trajectories was performed using the ULamDyn program.^52^ All electronic structure calculations were performed using the software Orca 5.0.^53^ One-electron transition density matrix (1-TDM) analysis was performed with the TheoDORE 3.2 package,^35^ enabling statistical analysis of the molecular electronic structures throughout the trajectories propagation. A classification of the character of electronic transitions is derived from it through a series of descriptors, which are formally defined in the .

## Supplementary Material







## Data Availability

Dataset containing the initial conditions and surface hopping dynamics is available at the Zenodo repository (DOI: 10.5281/zenodo.18456089).

## Notes

SI-HiddenRoutes: Tetramers theoretical spectra, NTOs of main absorption, trajectories final times, formal definition of electronic structure descriptors, descriptors distribution for sampled time steps, and Cartesian coordinates of optimized geometries (PDF)
Trajectories-movies: MP4 videos of example dynamics (ZIP)
Transparent Peer Review report available (PDF)
