# Supplementary material for: The Hidden Routes of DNA Photostability: Charge and Proton Transfer in Excited Cytosine–Guanine Tetramers
Source: J Phys Chem Lett. 2026 May 7;17(20):5709–17. doi: 10.1021/acs.jpclett.6c00376 (PMC13200244; doi:10.1021/acs.jpclett.6c00376)
Supplement: Supplementary file 1 [file jz6c00376_si_001.pdf]

Supporting Information

The Hidden Routes of DNA Photostability: Charge and Proton Transfer in Excited Cytosine-Guanine Tetramers

J. G. de Abrantes[1], J. M. Toldo[2,3], M. Barbatti[3,4], and M. Sacchi[1]

[1]*School of Chemistry and Chemical Engineering, University of Surrey, Guildford, GU2 7XH, United Kingdom*
[2]*Lyon 1 Université, ENS de Lyon, CNRS, Laboratoire de Chimie, UMR 5182, 69342 Lyon Cedex 07, France*
[3]*Aix Marseille University, CNRS, ICR, Marseille, France*
[4]*Institut Universitaire de France, 75231 Paris, France*

Contents

1 Absorption spectra **2**

2 NTOs of vertical excitations **2**

3 Final times of FSSH trajectories **4**

4 GC base pair benchmarking **4**

5 Electronic structure analysis of excitations – 1-TDM Descriptors **5**

6 Electronic structure analysis of the excitations – Results **6**

7 Benchmark of level of theory **8**

8 Cartesian coordinates **9**

References **11**

1 Absorption spectra

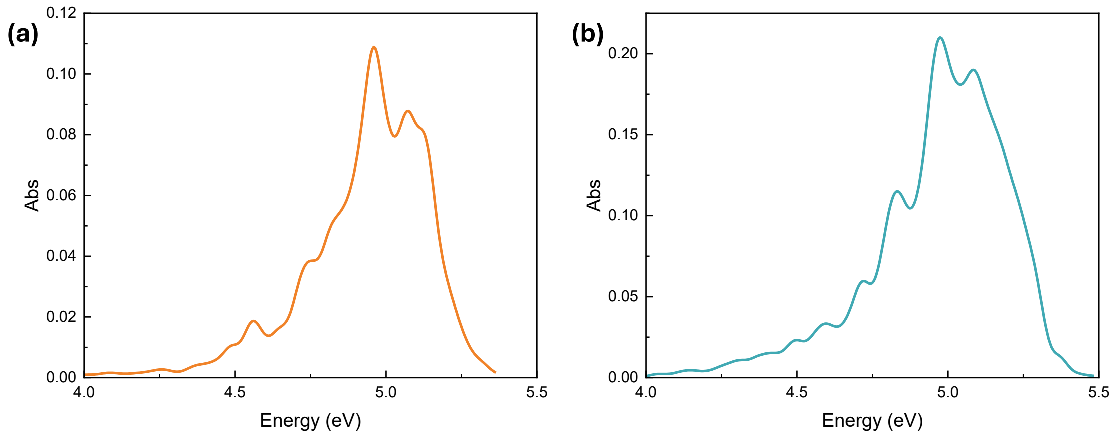

Figure S1: Nuclear ensemble absorption spectra obtained from the three first excited states for (a) non-alternating and (b) alternating tetramers.

2 NTOs of vertical excitations

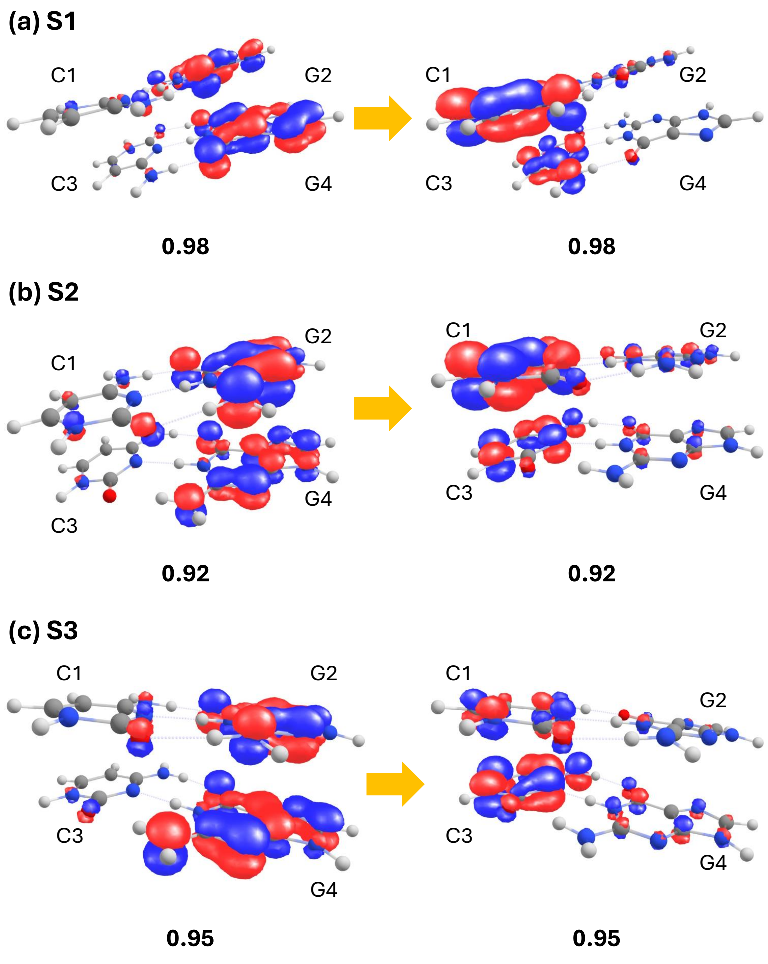

Figure S2: NTOs representing the vertical excitations of (a) S_1, (b) S_2 and (c) S_3 in the Franck-Condon region for the non-alternating tetramer. The absorption coefficients are shown in bold below each figure.

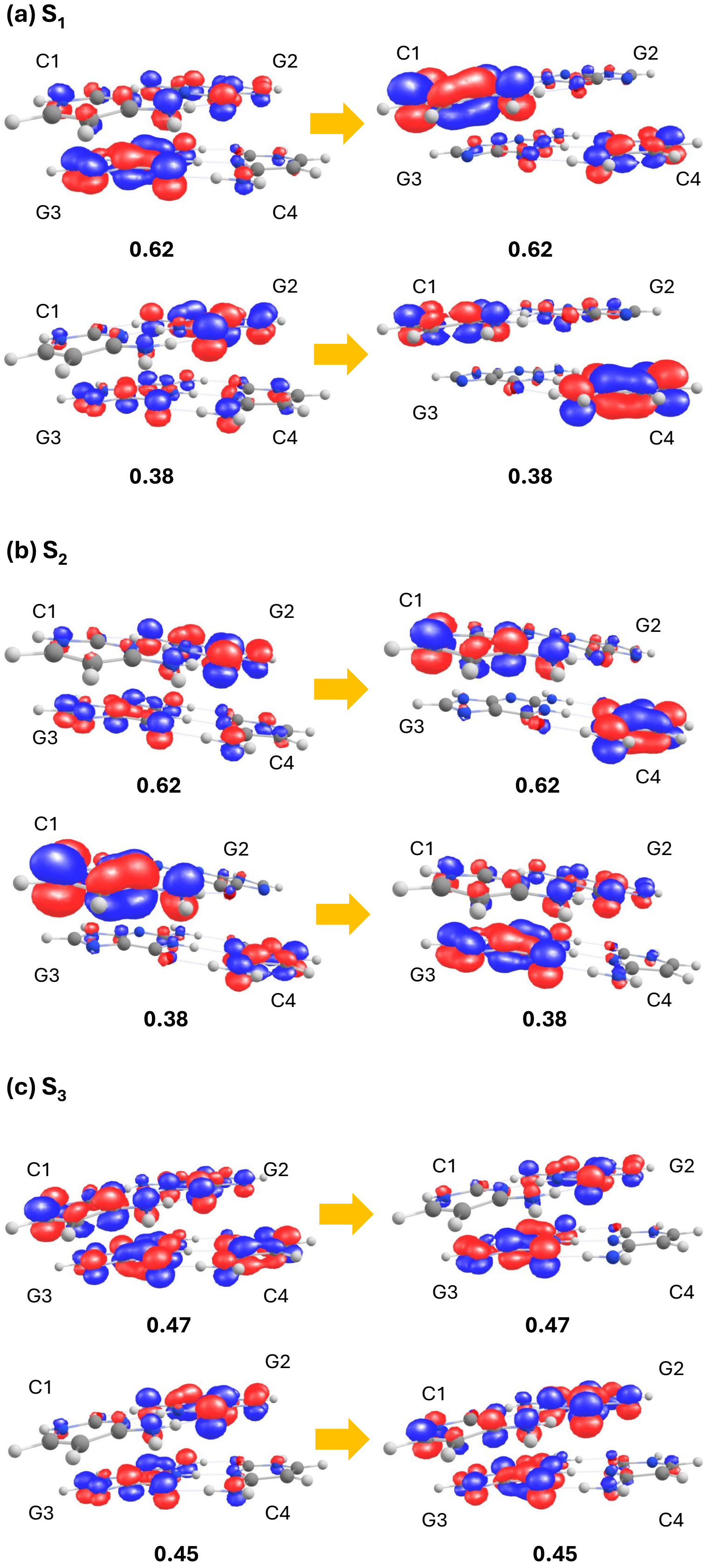

Figure S3: NTOs representing the vertical excitations of (a) S_1, (b) S_2 and (c) S_3 in the Franck-Condon region for the alternating tetramer. The absorption coefficients are shown in bold below each figure.

3 Final times of FSSH trajectories

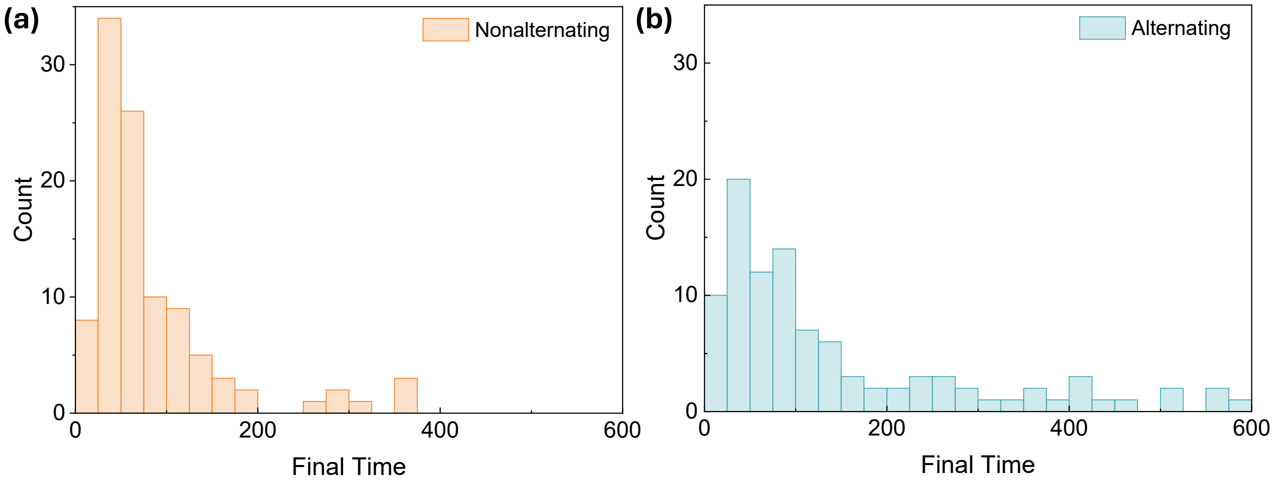

Figure S4: Final trajectory times reached in the excited state before finding a conical intersection to S_0 for (a) non-alternating and (b) alternating tetramers.

4 GC base pair benchmarking

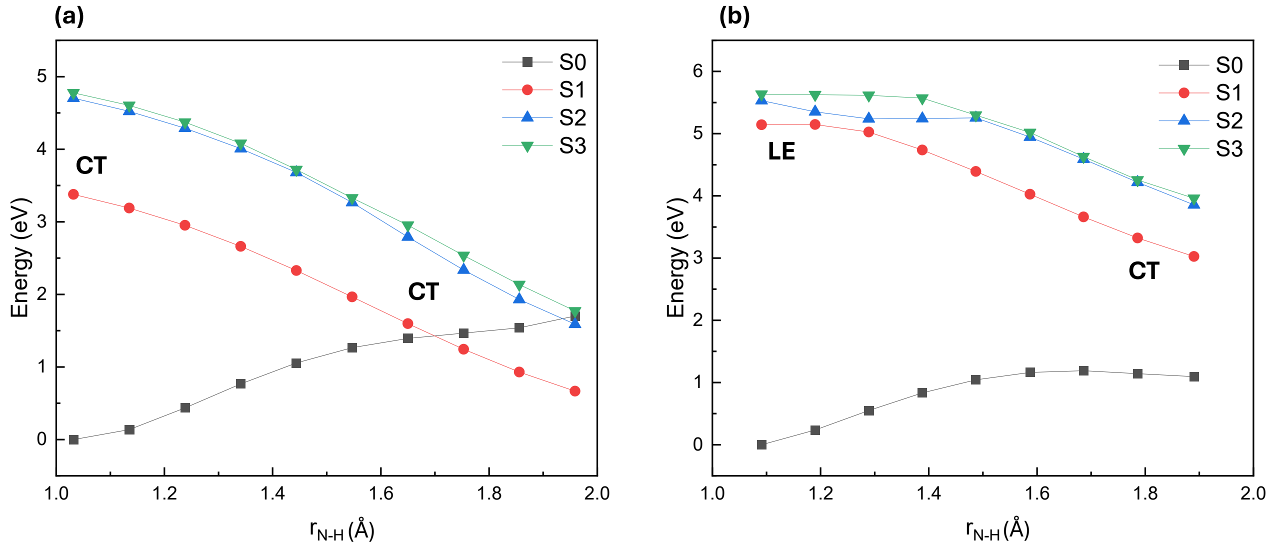

Figure S5: S_0, S_1, S_2 and S_3 potential energy surfaces of the GC base pair using TDDFT at the CAM-B3LYP/6-31G* level of theory, calculated through a linear interpolation between the canonical structure to the proton-transferred one, in the central N-H coordinate. Panel (a) starts from the optimised GC base pair in the S_0, whereas panel (b) starts from a base pair geometry generated by Wigner sampling.

5 Electronic structure analysis of excitations – 1-TDM Descriptors

The excited state classification was performed with the aid of the TheoDORE package [1, 2], where descriptors were extracted from the one-electron transition density matrix (1-TDM) of the system, defined as [3]

$$D^{IJ}_{\mu\nu} = \langle \Psi^I | \hat{a}^\dagger_\mu \hat{a}_\nu | \Psi^J \rangle$$

where I and J are two electronic states and μ and ν are orbital indices.

Then, a population analysis of the 1-TDM is performed, where the excitation is seen as an electron and hole or, in other words, an exciton is described by the use of a two-body wavefunction [3], $\chi_{exc}(r_H, r_E)$, where r_H and r_E are the coordinates of hole and electron, respectively.

For this purpose, the system was divided into fragments, and the excitations were analysed in terms of their orbital contribution. In this work, each nucleobase corresponds to a fragment, and the contribution from the orbitals in fragments A and B are given by [4]

$$\Omega_{AB} = \frac{1}{2} \sum_{\mu \in A} \sum_{\nu \in B} ((S^{1/2} D S^{1/2})_{\mu\nu})^2$$

where **S** is the overlap matrix between the two wave functions [3]

$$S_{\mu\nu} = \int \chi_\mu(r) \chi_\nu(r) dr.$$

From this analysis, it is possible to extract quantitative measures for the charge transfer character of the excitation[4]. The CT descriptor is defined as

$$CT = \Omega^{-1} \sum_{A, B \neq A} \Omega_{AB}.$$

The average delocalisation of the exciton (PR descriptor) is given by the average of participation ratios of electron and hole

$$PR_e = \frac{\Omega^2}{\sum_A (\sum_B \Omega_{AB})^2}$$

$$PR_h = \frac{\Omega^2}{\sum_B (\sum_A \Omega_{AB})^2}.$$

The participation ratio of NTOs, that accounts for the number of NTOs required to describe the excitation is given by [3]

$$PT_{NTO} = \frac{\Omega}{\sum_i \lambda_i^2}$$

where λ_i is the weight of the respective configuration.

Finally, the POS descriptor is given by the average taken between the position of electron and hole

$$POS_h = \Omega^{-1} \sum_A A (\sum_B \Omega_{AB})$$

$$POS_e = \Omega^{-1} \sum_B B (\sum_A \Omega_{AB}).$$

6 Electronic structure analysis of the excitations – Results

Panels a and b in figure S6 show the participation ratio of electron and hole, or e/h delocalisation (PR descriptor) versus the charge transfer character (CT descriptor) for sampled timesteps in all the surface hopping trajectories of (a) non-alternating and (b) alternating tetramers. This is a similar representation as Figure 6 in the main text, but the samples are coloured according to the PR_{NTO} descriptor instead, that expresses the resonant character of the excitation. For the analysis in this work, any structure with $PR_{NTO} > 1.25$ was considered to present resonant character, distinguishing local excitations from excitons and charge transfer from charge resonance states.

Panels c and d show the participation ratio of the natural transition orbitals (PR_{NTO}) of the excitation computed at specific sample times throughout the trajectories. Note that for charge transfer states ($CT > 0.8$), only a few points in the non-alternating tetramer present $PR > 1.25$. This indicates that very seldom charge resonant states were present in the dynamics.

Panels e and f show the correspondence of the number of entangled states (Z_{HE}) and PR_{NTO}. The higher number of configurations needed to describe an excitation (PR_{NTO}), the higher correlation degree between electron and hole. Observe that all points above 50 fs lie in the $PR_{NTO} < 1.25$, $Z_{HE} < 1.2$ region, meaning that, during propagation of trajectories in the excited state, the states lose their coherent character.

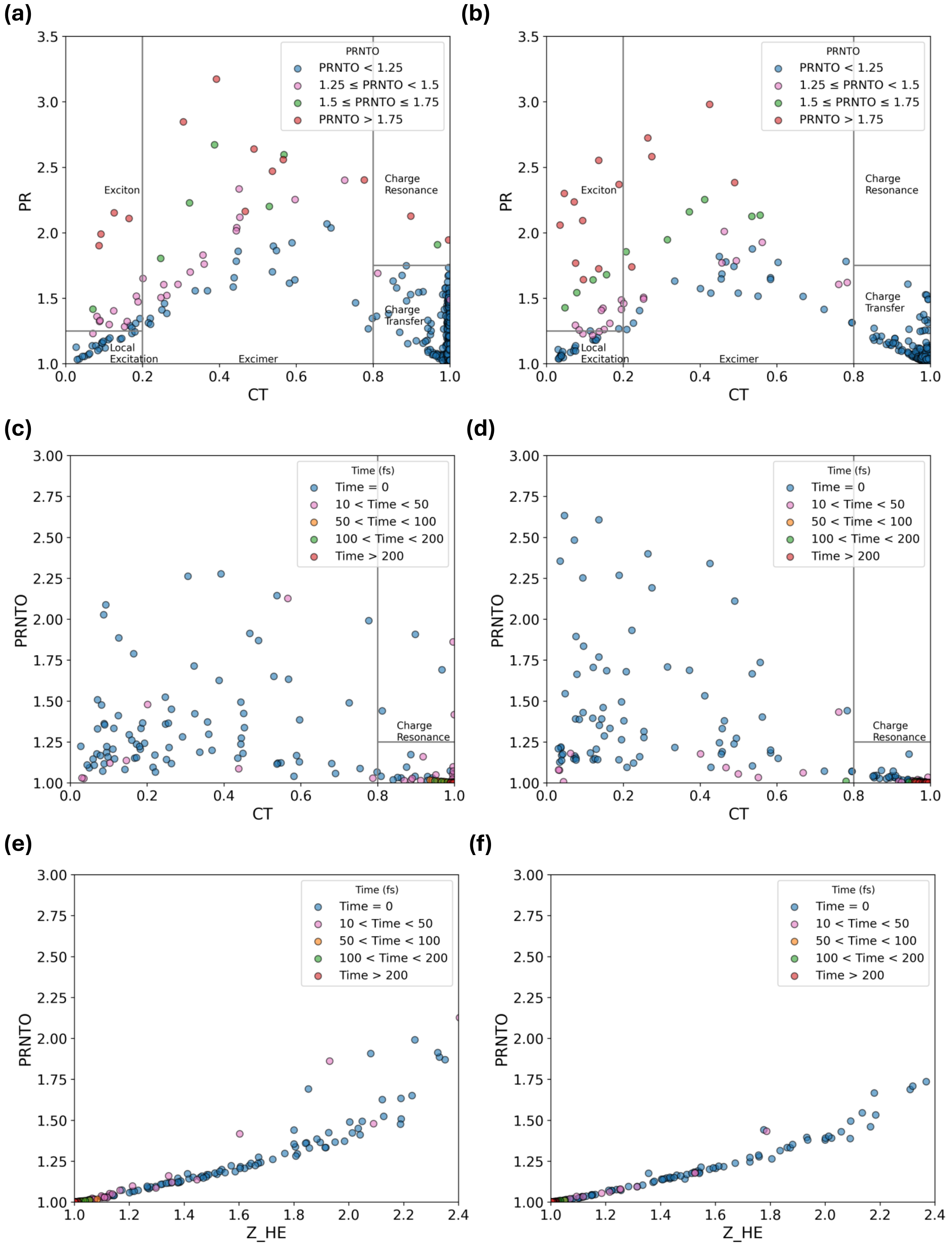

Figure S6: Panels a and b show the delocalisation of electron and hole versus charge transfer character for the non-alternating and alternating tetramers, respectively. The number of NTOs required to describe an excitation as a function of the CT character is shown in panels b and c for the non-alternating and alternating tetramers, respectively, and as a function of entangled states in panels e and f.

7 Benchmark of level of theory

In order to verify if the chosen level of theory used throughout the dynamics propagation would result in accurate energies and oscillator strengths, we have calculated the vertical excitations of the isolated nucleobases using the CAM-B3LYP functional and 6-31G* basis set and compared to the reference methods provided by Wiebeler *et al* [5] using multi reference methods. For guanine, SC-NEVPT2 is defined as the reference method, whereas for cytosine MRCISD+Q is chosen. Tables T1 and T2 show the first eight vertical excitations for guanine and cytosine, respectively.

Table T1: First eight vertical excitations for guanine, as calculated according to the method used throught this work (TDDFT, CAM-B3LYP and 6-31G* basis set) and compared to a multi-reference method (SC-NEVPT2). [5]

	TDDFT		SC-NEVPT2	
Character	Energy (eV)	Oscillator strength	Energy (eV)	Oscillator strength
$\pi\pi^*$	5.48	0.221	5.14	0.219
nπ^*	5.62	0.002	5.72	–
$\pi\pi^*$	6.08	0.313	5.98	0.133
nπ^*	6.46	0.000	6.73	–
nπ^*	6.60	0.006	–	–
nπ^*	7.06	0	–	–
$\pi\pi^*$	7.11	0.001	6.87	0.023
$\pi\pi^*$	7.23	0.006	7.20	0.225

Table T2: First eight vertical excitations for cytosine, as calculated according to the method used throught this work (TDDFT, CAM-B3LYP and 6-31G* basis set) and compared to a multi-reference method (MRCISD+Q). [5]

	TDDFT		MRCISD+Q	
Character	Energy (eV)	Oscillator strength	Energy (eV)	Oscillator strength
$\pi\pi^*$	5.31	0.074	4.89	0.059
nπ^*	5.36	0.001	5.37	–
nπ^*	5.94	0.001	5.70	–
nπ^*	6.17	0.000	–	–
$\pi\pi^*$	6.31	0.133	5.95	0.140
nπ^*	6.92	0.001	–	–
$\pi\pi^*$	7.34	0.245	7.08	0.882
$\pi\pi^*$	7.43	0.000	7.35	0.183

Note that Wiebeler *et al* [5] only computed two $n\pi^*$ singlet states, whereas our method allows for any number of $n\pi^*$ and $\pi\pi^*$ excitations, reason why some of the reference energy values are missing on the table. Likewise, given that their calculations used C_s symmetry, the oscillator strengths for $n\pi^*$ cannot be computed.

Although the vertical excitations do not coincide exactly in energy value, the difference remains below 0.34 eV for guanine and 0.42 eV for cytosine. However, given that the trends of the ordering of the states and the oscillator strengths are analogous for both methods, we conclude that the method chosen correctly reproduces the trends in energy and populated states throughout the dynamics propagation.

8 Cartesian coordinates

The cartesian coordinates of the optimised ground state for the non-alternating tetramer are given below in Å.

H	-1.82844893607196	1.13906787314558	1.68325742812203
N	-1.11147000302126	0.66432999866556	2.21153000076984
C	0.20288540355643	0.80799233727105	1.72424206833411
O	0.37523803112717	1.45301177741940	0.69092917991903
N	1.19773793241969	0.22183150206196	2.43003993369726
C	0.93377402293089	-0.54192071294211	3.48913492684469
N	1.95355374221796	-1.09874626645423	4.13084986685797
C	-0.42034558759963	-0.77113272341891	3.93744449634903
C	-1.40382639825063	-0.12792884408158	3.27535883567561
H	-2.44966746423631	-0.20388787030618	3.55059477236641
H	-0.63114469479628	-1.39733948528687	4.79411132981741
H	2.93329657230551	-0.89799566542369	3.85705738902022
H	1.79297142724051	-1.67774831070828	4.93943962937503
H	0.10294307728589	-0.06271250629400	-2.36807055756380
N	0.30713000216706	-0.73917000921117	-1.64704999904579
C	1.66084951154791	-0.83306974487197	-1.27780876922859
O	2.48547110716566	-0.17123094021967	-1.90370952835543
N	1.97308036289067	-1.66966004768152	-0.25799814692181
C	1.02408282177482	-2.31199402784076	0.41500506349881
N	1.39446593764213	-3.11415683678636	1.41147184355875
C	-0.37347790202954	-2.15880889394692	0.09286662922917
C	-0.67610585639514	-1.35274404674248	-0.94651023589557
H	-1.69198544177322	-1.16716556659680	-1.27630300604438
H	-1.14086885858626	-2.67251599333583	0.65629609434878
H	2.39035077422854	-3.19492721449222	1.69271093976132
H	0.69692131740023	-3.60496602147328	1.94649331646962
H	9.39568042446462	-2.10059233646989	1.38721195497516
N	8.52537002470298	-2.50422022856126	1.69349002789468
C	8.29803501869193	-3.28008378140702	2.81287139797251
N	7.04415773250915	-3.56033091318983	2.99343408363137
C	6.39545823569831	-2.92243241591556	1.95614201949567
C	5.00762594415856	-2.83738255000809	1.63579998830228
O	4.04367027171023	-3.34800794157773	2.21533114409405
N	4.79855737044316	-2.05456919037400	0.49533137828045
C	5.77337764563809	-1.43074401943122	-0.23769079578259
N	5.34681691853966	-0.73356581328726	-1.31624599904664
N	7.05592609023731	-1.50164584493913	0.04648211568829
C	7.29900117930605	-2.25095534846055	1.14248630763306
H	9.11191832685533	-3.60966373527546	3.44412966881585
H	3.81557893162189	-1.91870619801246	0.21681452713754
H	6.01712896147776	-0.09010207058733	-1.70526751172513
H	4.35726692350622	-0.52484809462226	-1.45990848814721
H	8.27886779220416	2.25807543546629	0.39964917024153
N	7.80295996782611	1.74145023753722	1.12212997288575
C	8.36223121211365	1.02474212014433	2.15989060952217
N	7.47946976788640	0.43408714342291	2.90576111799357
C	6.26839174211104	0.76850329869096	2.33561132396710
C	4.93906307309320	0.37546773529576	2.67519688622952
O	4.56711503974596	-0.37214896223797	3.58096922375809
N	4.00106360029045	0.94323666303148	1.80510566004320
C	4.28942868075247	1.75706819204322	0.74224628628347
N	3.22809411613501	2.23556383793822	0.05794418969713
N	5.51489959259692	2.09636131793175	0.39563010691690
C	6.44746989058434	1.57750681212168	1.22100078470381
H	3.01843457619226	0.68699378932154	1.98158416456486
H	3.43212424652797	2.62931899678249	-0.84645792884647
H	2.28997689197713	1.86651831589772	0.20946727243386
H	9.43305574249723	0.99531296072898	2.31098595676338

The cartesian coordinates of the optimised ground state for the alternating tetramer are given below in Å.

N	-0.99554003896447	-4.21337010989446	2.93546995539146
H	-1.73436776235576	-3.92503584858679	3.55918203769106
C	0.29558877049064	-4.19152891304489	3.48514250173473
O	0.42286613211520	-3.88864070605729	4.67248787382338
N	1.33029250286881	-4.52470121443574	2.67421505092023
C	1.13309085610562	-4.73803518181922	1.37720341034118
N	2.18007295795496	-5.09722488766028	0.63050199445887
C	-0.17316773307694	-4.64166761072102	0.78042309719299
C	-1.20530397345507	-4.37212246447012	1.60322367290885
H	-2.22903685707188	-4.28107085670809	1.25984544928878
H	-0.31686297149507	-4.77915840452445	-0.28252315480313
H	3.13389274277915	-5.02959957177381	1.01341650067283
H	2.08223298932486	-5.08141209577792	-0.37200288831828
H	0.24701119324764	-0.05929390818911	5.31802143781593
N	0.49844003204211	-0.40871988708316	4.40691004601268
C	-0.34203344609351	-0.79810598865249	3.38735620734857
N	0.28994853569938	-1.27910547605362	2.35845257462377
C	1.62394630006272	-1.21617304960851	2.71322687736704
C	2.80071295434494	-1.60064303950260	1.99854924880372
O	2.88798480137675	-2.09304843053945	0.86996275623962
N	3.95370534709383	-1.35289659974627	2.74735433914526
C	3.98499508649390	-0.84104522576269	4.01908566256261
N	5.20390688422483	-0.69554447737329	4.56801104654590
N	2.90482967376125	-0.48832730282950	4.69096263278035
C	1.77393046100495	-0.69150829407479	3.99045131377486
H	-1.41482858970272	-0.68499649594002	3.46861818286370
H	4.85234059637630	-1.59632006658649	2.29781689218183
H	5.22696956219460	-0.41938169251202	5.53553724835634
H	6.06745503601394	-1.01441030755432	4.11846439443406
H	9.70546390862927	-2.19288888145059	2.12015315212026
N	8.87724997163270	-2.26858006413690	1.54894010346028
C	7.67669305950293	-1.91450729393416	2.18437146160892
O	7.72761154277401	-1.48951818163296	3.33932289490758
N	6.52707702712176	-2.04656435745130	1.47716571988567
C	6.53375444535725	-2.60815008574288	0.27240750461629
N	5.37936263920495	-2.68244573648954	-0.39469298411897
C	7.74721106239462	-3.08347305273134	-0.33869352600485
C	8.89331302411974	-2.89381379922945	0.34397115181148
H	9.86196251226807	-3.20994416793466	-0.02488524075485
H	7.73674707749419	-3.57205725583046	-1.30337228998870
H	4.49246494748582	-2.47884238634682	0.08750732428387
H	5.33711116588978	-3.25549287259357	-1.22203320093234
H	8.16759653928319	-4.25482500107664	6.02784987580448
N	7.77649002769202	-4.47521993979872	5.12567989557258
C	8.44868104833715	-4.78416221883263	3.96364260935367
N	7.66007019678502	-4.94195133646835	2.94250606142765
C	6.39352317258225	-4.71674298679515	3.44771145799869
C	5.11369748791211	-4.74517714990442	2.81160214749306
O	4.84750180329011	-4.98214626001672	1.62982461307407
N	4.08925409411254	-4.45961190859525	3.71754850788551
C	4.25879320275308	-4.14559515128561	5.04163529958983
N	3.13846910532567	-3.87384632471966	5.73233060856465
N	5.43600899733866	-4.10784495675289	5.63779023695788
C	6.44633762184767	-4.40819455833560	4.80092588245440
H	9.52518790168220	-4.88993911975150	3.94834820928364
H	3.12622609364986	-4.47544415237829	3.34211918367672
H	3.25880826699113	-3.57828767603649	6.68658405411362
H	2.20194490983002	-3.86908039303049	5.31714083933273

References

[1] Felix Plasser and Hans Lischka. “Analysis of Excitonic and Charge Transfer Interactions from Quantum Chemical Calculations”. en. In: *Journal of Chemical Theory and Computation* 8.8 (Aug. 2012), pp. 2777–2789.

[2] F. Plasser. “TheoDORE: A toolbox for a detailed and automated analysis of electronic excited state computations”. In: *The Journal of Chemical Physics* 152.8 (2020), p. 084108.

[3] Felix Plasser, Michael Wormit, and Andreas Dreuw. “New tools for the systematic analysis and visualization of electronic excitations. I. Formalism”. In: *The Journal of Chemical Physics* 141.2 (July 2014), p. 024106.

[4] Lea M. Ibele et al. “Excimer Intermediates en Route to Long-Lived Charge-Transfer States in Single-Stranded Adenine DNA as Revealed by Nonadiabatic Dynamics”. en. In: *The Journal of Physical Chemistry Letters* 11.18 (Sept. 2020), pp. 7483–7488.

[5] Christian Wiebeler et al. “Excitation energies of canonical nucleobases computed by multiconfigurational perturbation theories”. In: *Photochemistry and photobiology* 93.3 (2017), pp. 888–902.
