# Supplementary material for: The Hidden Routes of DNA Photostability: Charge and Proton Transfer in Excited Cytosine–Guanine Tetramers
Source: J Phys Chem Lett. 2026 May 7;17(20):5709–17. doi: 10.1021/acs.jpclett.6c00376 (PMC13200244; doi:10.1021/acs.jpclett.6c00376)
Supplement: Supplementary file 3 [file jz6c00376_si_003.pdf]

jz-2026-00376u.R1

Name: Peer Review Information for "The Hidden Routes of DNA Photostability: Charge and Proton Transfer in Excited Cytosine-Guanine Tetramers"

First Round of Reviewer Comments

Reviewer: 1

Comments to the Author

The manuscript presents a compelling and thorough investigation of proton transfer pathways in electronically excited guanine–cytosine systems. Charge and proton transfer processes triggered by light irradiation are topics of broad and current interest, extending well beyond strictly biological or bio-inspired contexts. The authors employ state-of-the-art electronic structure methods and nonadiabatic dynamics simulations, and the results are both impactful and scientifically sound. The paper is well written, the methodology is sound and appropriately implemented, the authors provide a comprehensive coverage of the relevant literature and include discussions and perspectives. Overall, I have very few comments that the authors may wish to consider before publication, as the manuscript is already in excellent shape.

(1) Given the technical level of the journal's readership, I would recommend specifying the functional and basis set at the very beginning of the manuscript. For instance, writing TD-CAM-B3LYP instead of the more general TD-DFT would improve clarity. It would also be helpful to explicitly state that the study is performed in the gas phase.

(2) The oscillator strengths reported in Table 1 are relatively low. I assume this is related to the charge-transfer (CT) character of the corresponding excited states. Could the authors provide a quantitative measure of the CT character for these transitions also there? Additionally, it would be interesting to comment on how the dynamical outcomes might differ if more "pure" ππ* excited states were initially populated (S4 or S5...).

(3) In the conclusions, the authors mention the possibility of using QM/MM approaches to account for solvent effects. Could they also comment on whether a similar strategy could be employed to extend the DNA model itself?

(4) A minor cosmetic suggestion: I recommend being consistent with the notation of electronic states (S_0, S_1, etc.) as subscripts throughout the manuscript, including in the figures.

Reviewer: 2

Comments to the Author

Sacchi and coworkers have investigated the photostability of two (GC)2 alternating and non-alternating topologies to unravel the population of S1, S2 and S3 states along non-adiabatic SH dynamics rooted in TDDFT (350 and 700ns). This investigation duly refers to previous studies and essentially leads to the same conclusions, evidencing two types of proton transfer after the initial charge separation and mostly occurring interstrand.

The authors then concisely delineate mechanistic scenarios for the proton transfer / charge which they observed along the non-adiabatic SH dynamics back to the ground state and quantify them along 200 trajectories, evidencing the impact of the (GC)2 topology on the intra- and "diagonal" electron transfer.

This Letter provides an accurate cartography of competing excitation pathways which completes very nicely previous works, and suggest relevant descriptors which are of interest of the readership of JPCL. The authors rightfully conclude that QM/MM simulations including the solvent are needed to take into account the actual embedding of the B-DNA helix, with also other base pairs prone to tune the photophysics of relaxation.

Reviewer: 3

Comments to the Author

Gonçalves de Abrantes et al have performed in this letter a quantum-chemistry study on the excited-state dynamics of a model of DNA based on two stacked guanine-cytosine base pairs. Although I am a bit concerned about the method that the authors use and the constraints of the molecular model, the manuscript reports interesting findings and electronic-structure analyses on the role of charge transfer and proton transfer which may be of interest for the community of researchers on DNA excited-state dynamics.

I consider that the authors should address the following points to provide higher reliability:

- Previous studies on dimers or multimers using higher-level methods (for instance, multireference methods) predict the charge transfer states appearing at much higher energies as compared to the localized states. Is this the case here? If not, is it associated to the deficiency of TDDFT? The discussion related to Table 1 indicates that the low-lying states are ππ* excitation from a π in guanine to a π* in cytosine. Later on, in Figure 6 I see local excitations at time zero. Can the authors clarify these aspects?

- I do not see any reference to nπ* states which shall also appear among the low-lying excited states. These states are S1 in the isolated nucleobases and recent works point to a key role in the non-radiative decay of nucleobases. Are they not found in the current calculations? Why?

- In this context, I am missing a benchmark of the level of theory used (TDDFT with CAM-B3LYP and 6-31G*) in the isolated nucleobases (cytosine and guanine) comparing with previous high-level calculations to demonstrate that the level of theory used here is correctly reproducing the energies and oscillator strengths of nπ* and ππ* states.

- The common knowledge on nucleobases is that DNA photostability arises primarily to localized non-radiative decay in single nucleobases via ring-puckering conical intersections. I do not see these path in the findings obtained by the authors. How this manuscript reconciles with such state-of-the-art in the field? The current study seems to point to proton transfer as fully responsible for photostability. May be this is due to the geometrical constraints used in this study (fixing carbon coordinates)?

- As the authors state at the end, the current model is not able to predict formation of pyrimidine dimers, which is an important lesion in DNA. Furthermore, I guess that excimer formation is neither properly described as the result of the geometrical constraints. Therefore, decay times are underestimated, is this right?

Author's Response to Peer Review Comments:

Thank you for giving us the opportunity to submit a revised draft of the manuscript "The Hidden Routes of DNA Photostability: Charge and Proton Transfer in Excited Cytosine-Guanine Tetramers". We thank all the reviewers for their reports. We appreciate their time and effort to provide insightful comments and suggestions. In light of the reviewers' concerns, we have revised the paper's content, incorporating all their suggestions. Please see in the document attached our responses and changes to the paper in line with their suggestions.

Response to reviewer reports

The Hidden Routes of DNA Photostability: Charge and Proton Transfer in Excited Cytosine-Guanine Tetramers

J. G. de Abrantes, J. M. Toldo, M. Barbatti, M. Sacchi

March 18, 2026

Thank you for giving us the opportunity to submit a revised draft of the manuscript "The Hidden Routes of DNA Photostability: Charge and Proton Transfer in Excited Cytosine-Guanine Tetramers". We thank all the reviewers for their reports. We appreciate their time and effort to provide insightful comments and suggestions. In light of the reviewers' concerns, we have revised the paper's content, incorporating all their suggestions.

We have highlighted in red the changes we have made in the manuscript to address the referees' comments. Outlined below are our responses and changes to the paper in line with their suggestions.

Our response is formatted in the following way:

The Reviewers' comments are in italics and coloured blue.

Response:

Our response is written unindented adjacent to the bold lettering.

The changes made to the revised manuscript are in red.

Reviewer 1

> *The manuscript presents a compelling and thorough investigation of proton transfer pathways in electronically excited guanine–cytosine systems. Charge and proton transfer processes triggered by light irradiation are topics of broad and current interest, extending well beyond strictly biological or bio-inspired contexts. The authors employ state-of-the-art electronic structure methods and nonadiabatic dynamics simulations, and the results are both impactful and scientifically sound. The paper is well written, the methodology is sound and appropriately implemented, the authors provide a comprehensive coverage of the relevant literature and include discussions and perspectives. Overall, I have very few comments that the authors may wish to consider before publication, as the manuscript is already in excellent shape.*

Response:

We thank Reviewer 1 for their interest in the manuscript and their cordial review. We are grateful for the positive comments regarding the quality of the writing and methodology. We now highlight the following key improvements made to the revised manuscript as motivated by the reviewer's comments: • Addition of the level of theory and conditions of calculations in the abstract and introductory part of the paper;

- Improvements on Table 1 and respective discussion in the main text;
- Additional suggestions on the conclusive part of the paper;
- Correction to the legend in Figure 2.

Point 1

> *Given the technical level of the journal's readership, I would recommend specifying the functional and basis set at the very beginning of the manuscript. For instance, writing TD-CAM-B3LYP instead of the more general TDDFT would improve clarity. It would also be helpful to explicitly state that the study is performed in the gas phase.*

Response:

Following the reviewer's suggestion, we have incorporated the functional description and the fact that the studies were carried out in the gas phase, both in the Abstract (lines 6-8 in the manuscript) and the main text (lines 45-47).

In the abstract, page 2, the text now reads:

Using nonadiabatic surface-hopping dynamics at the TD-CAM-B3LYP level, we investigate the excited-state behaviour of DNA tetramers composed of stacked guanine–cytosine $(GC)_2$ dimers in alternating and non-alternating sequences in the gas phase.

In the main text, page 3, lines 45-47, now read:

With this in mind, in this study we performed surface hopping dynamics using linearresponse time-dependent density functional theory (TDDFT) using the CAM-B3LYP functional in tetramers composed of two-stacked base pairs of guanine and cytosine nucleobases in the gas phase.

Point 2

> *The oscillator strengths reported in Table 1 are relatively low. I assume this is related to the charge-transfer (CT) character of the corresponding excited states. Could the authors provide a quantitative measure of the CT character for these transitions also there? Additionally, it would be interesting to comment on how the dynamical outcomes might differ if more "pure" ππ∗ excited states were initially populated (S4 or S5...).*

Response:

(1) Oscillator strengths. As the reviewer pointed out, the low oscillator strengths reflect the small overlap integrals between the initial and final states upon absorption, as captured by the high CT character, which we have now included as an extra column in Table 1. Besides the high CT number, for the alternating tetramer, the PR_{NTO} parameter is > 0.25 upon absorption in the Franck-Condon region, meaning that more than one NTO is necessary to describe the excitation. Therefore, while the alternating tetramer can be classified as a charge-transfer state at the equilibrium geometry in the Franck-Condon region, the non-alternating is better classified as a charge resonance state. For better visualisation of the excitation character, we have split Figure S2 into two figures (S2 and S3) in the supporting information to include the NTOs of vertical excitations for all three states, S_1, S_2, and S_3. Besides the new column including the CT character in Table 1, the respective discussion in the main text now reads (page 4, line 60):

The high charge (CT) transfer character number (GG→CC) shown in Table 1 (and further discussed in connection with Figure 5), reflects the small overlap integrals between initial and final states upon absorption, explaining the low oscillator strengths. The alternating tetramer requires more than one orbital transition to describe the excitations and presents PR_{NTO} parameter of 0.25 (as further discussed); therefore, they are better classified as a charge resonance state.

(2) Population of bright states. We thank the reviewer for raising this point. Higher-lying and "brighter" states were not included in the present simulations,but we agree that their population could, in principle, introduce additional relaxation channels or alter the subsequent S_1 dynamics. For this reason, the conclusions of the present work are restricted to the excitation regime considered here, which is the lowest bright state. We have clarified this point in the revised manuscript in page 5, line 86:

Therefore, the relaxation channels reported in this work are restricted to this excitation regime (i.e., excitation to the lowest bright state), being that if more bright $\pi\pi*$ states were initially populated, the observed dynamics might be affected.

Point 3

In the conclusions, the authors mention the possibility of using QM/MM approaches to account for solvent effects. Could they also comment on whether a similar strategy could be employed to extend the DNA model itself?

Response:

The fragment-based analysis of the electronic character used in this study can be used for results obtained with QM/MM, to the extent that is included in the QM region. Despite the evidence that the backbone is not directly involved in the photochemical processes, [1] the inclusion of a section of the DNA backbone in the MM region might affect the development of the dynamics by modulating the conformational interplay of the nucleobase chromophores, which, in turn, might affect the charge distribution during the dynamics. Nevertheless, to be included in the fragment-based analysis done in this paper, the backbone (or other nucleobases) need to be included in the QM region. This fact has now been made explicit in the conclusion, page 11, line

284:

Under the QM/MM framework, the explicit consideration of the DNA backbone in the MM region could modulate the conformational interplay of the nucleobase chromophores, [1] which, in turn, might affect the charge distribution during the dynamics and enable

outcomes such as cyclobutane pyrimidine dimer (CPD) formation to be assessed. It should be taken into consideration, however, that given that the formation of CPDs is a relatively rare event, [2] running a statistically relevant number of trajectories would be required. We highlight that it is possible to use the fragmentbased approach to follow the charge evolution also in the QM/MM framework, as long as the fragments to be considered are included in the QM region.

Point 4

> *A minor cosmetic suggestion: I recommend being consistent with the notation of electronic states (S_0, S_1, etc.) as subscripts throughout the manuscript, including in the figures.*

Response:

We thank the reviewer for bringing this inconsistency to our attention. The notation has now been updated in Figure 2 on the main manuscript.

Reviewer 2

> *Sacchi and coworkers have investigated the photostability of two (GC)2 alternating and non-alternating topologies to unravel the population of S1, S2 and S3 states along non-adiabatic SH dynamics rooted in TDDFT (350 and 700 ns). This investigation duly refers to previous studies and essentially leads to the same conclusions, evidencing two types of proton transfer after the initial charge separation and mostly occurring interstrand. The authors then concisely delineate mechanistic scenarios for the proton transfer / charge which they observed along the non-adiabatic SH dynamics back to the ground state and quantify them along 200 trajectories, evidencing the impact of the (GC)2 topology on the intra- and "diagonal" electron transfer. This Letter provides an accurate cartography of competing excitation pathways which completes very nicely previous works, and suggest relevant descriptors which are of interest of the readership of JPCL. The authors rightfully conclude that QM/MM simulations including the solvent are needed to take into account the actual embedding of the B-DNA helix, with also other base pairs prone to tune the photophysics of relaxation.*

Response:

We thank Reviewer 2 for their evaluation of our manuscript and for their overall positive assessment. We are grateful for their clear recommendation that the work is suitable for JPCL.

Reviewer 3

Gon¸calves de Abrantes et al have performed in this letter a quantumchemistry study on the excited-state dynamics of a model of DNA based on two stacked guanine-cytosine base pairs. Although I am a bit concerned about the method that the authors use and the constraints of the molecular model, the manuscript reports interesting findings and electronic-structure analyses on the role of charge transfer and proton transfer which may be of interest for the community of researchers on DNA excited-state dynamics. I consider that the authors should address the following points to provide higher reliability:

Response:

We thank Reviewer 3 for their interest in the manuscript and detailed review, which we believe raises important discussions. We will now address the reviewer's comments point by point, highlighting the following key improvements made to the revised manuscript:

- Inclusion of discussion about ordering of CT and LE states on the manuscript; • Explanation about the presence of LE and other states at time = 0 in Figure 6;
- Discussion about the absence of low-lying $n\pi*$ states;
- Inclusion of a benchmark for the isolated nucleobases in the Supporting Information, comparing the TDDFT method used in this work to multi-reference ones reported in literature;
- Discussion about the implications of system applied constraints;
- Comment about CPD formation in the conclusive part.

Point 1

Previous studies on dimers or multimers using higher-level methods (for instance, multireference methods) predict the charge transfer states appearing at much higher energies as compared to the localized states. Is this the case here? If not, is it associated to the deficiency of TDDFT? The discussion related to Table 1 indicates that the low-lying states are $\pi\pi*$ excitation from a π in guanine to a $\pi*$ in cytosine. Later on, in Figure 6 I see local excitations at time zero. Can the authors clarify these aspects?

Response:

The authors thank the reviewer for this important comment. We agree that our ordering of the low-lying states may differ from that reported in other studies. For example, for a B-DNA-like GCGC tetramer computed at the ADC(2) level using ideal B-helix tetramer geometries in vacuo, Plasser *et al.* [3] found that the lowest chargetransfer state lays above several lower-lying localised $\pi\pi*$ states rather than among the lowest excited states. In the present work, this is not the ordering obtained at the TD-CAM-B3LYP level. As shown in the revised Table 1, the lowest vertical states of our tetramers display strong GG→CC CT character according to the fragmentbased CT descriptor. We have therefore revised the manuscript to make explicit that our state ordering differs from that reported by Plasser *et al.* and should be interpreted with appropriate caution. At the same time, we prefer not to attribute this difference to a single cause. In addition to the different electronic-structure methods, the two studies also differ substantially in their structural models. Plasser *et al.* considered exclusively stacked bases along one strand in the QM region, whereas our work examines double-helix-like tetramers. We therefore believe it is safer to describe the discrepancy as arising from a combination of method and model differences rather than attributing it solely to a generic deficiency of either TDDFT or ADC(2). The following changes have been made to the text, page 4, line 65, to highlight these discrepancies:

Depending on the method and geometry used, locally excited states might be found lying lower in energy than states with strong CT character. [4] In a study using ACD(2), where four CGCG stacked bases along one strand are treated in the QM region, Plasser *et al.* [3] found the first CT state to be S_{17}. Therefore, the ordering of states must be considered *cum grano salis*, being that it is highly sensitive to the method and geometry chosen.

In this context, Figure 6 provides an important clue. Table 1 characterizes the vertical excitations at the equilibrium geometry, whereas Figure 6 reflects the states sampled from the Wigner-sampled ensemble used to initiate the dynamics. As discussed in the manuscript, the Wigner sampling introduces small distortions around the equilibrium structure, yet these are already sufficient to produce a broad distribution of local, excitonic, mixed, and CT characters at time zero. This indicates that the low-lying excited-state manifold is highly sensitive to small geometry variations, reinforcing the importance of the structural model. In other words, the large spread of characters in Figure 6 suggests that modest geometry differences can alter both state character and state ordering. Thus, the broad distribution at t = 0 does not contradict Table 1. Rather, it indicates that the equilibrium-geometry picture of Table 1 is only one reference point within a low-energy manifold that is strongly affected by small structural distortions. This clarification has now been added to the manuscript in page 9, line 217:

The generation of initial conditions through a Wigner distribution creates an ensemble of structures slightly distorted from the equilibrium geometry. This distortion, combined with the high density of nearly degenerate excited states, may cause the initially accessed states to span different characters. As shown in Figure 6, the electronic populations are dominated by Local excitation, Excitonic resonance (exciton), and Mixed LE+CR (excimer) character. This provides evidence of how sensitive the electronic structure is to the geometric conformation.

Point 2

I do not see any reference to $n\pi*$ states which shall also appear among the low-lying excited states. These states are S1 in the isolated nucleobases and recent works point to a key role in the non-radiative decay of nucleobases. Are they not found in the current calculations? Why?

Response:

While we acknowledge that $n\pi^*$ states provide a different route for the decay pathway and they may be important for isolated nucleobases, recent benchmark studies on the lowest singlet excitations on guanine and cytosine [5] place $\pi\pi^*$ states as the lowest singlet excited states. In the tetramer, these nucleobase-centered $\pi\pi^*$ excitations generate a band of low-energy states that dominates the bottom of the excited-state spectrum. This comment has been added to the manuscript, page 4, line 79:

In isolated guanine and citosine, $\pi\pi^*$ states are the lowest singlet excited states. [5] Thus, in the tetramer, these nucleobase-centered $\pi\pi^*$ excitations generate a band of low-energy states that dominates the bottom of the excited-state spectrum, as previously reported. [6,7]

Point 3

In this context, I am missing a benchmark of the level of theory used (TDDFT with CAM-B3LYP and 6-31G) in the isolated nucleobases (cytosine and guanine) comparing with previous high-level calculations to demonstrate that the level of theory used here is correctly reproducing the energies and oscillator strengths of $n\pi*$ and $\pi\pi*$ states.*

Response:

As required by the reviewer, we have included a benchmark for the nucleobases comparing the method used throughout this work with higher theory levels, as showed by Wiebeler *et al.*, [5] as **Item 6** in the supporting information. As the trends of the ordering of the states and the oscillator strengths are analogous for both methods, we conclude that the method chosen is fit to reproduce the trends in energy and populated states throughout the dynamics propagation.

Point 4

> *The common knowledge on nucleobases is that DNA photostability arises primarily to localized non-radiative decay in single nucleobases via ringpuckering conical intersections. I do not see these path in the findings obtained by the authors. How this manuscript reconciles with such stateof-the-art in the field? The current study seems to point to proton transfer as fully responsible for photostability. May be this is due to the geometrical constraints used in this study (fixing carbon coordinates)?*

Response:

(1) Relative accessibility of puckering vs proton-transfer seams. In our study, both proton-transfer and ring-puckering internal conversion pathways are energetically accessible in the tetramers. However, the dynamics preferentially follow proton transfer because it is reached with a smaller structural displacement. In the monomer dynamics schematic of Barbatti *et al.*, [8] access to the S1/S0 crossing region occurs after displacements along the mass-weighted coordinate of roughly ˜3 ˚A·amu$^{1/2}$ for cytosine and ˜6 ˚A·amu$^{1/2}$ for guanine (see the reference paper Fig. 3). In our tetramers, the proton-transfer-associated seam is reached after a displacement usually < 1 ˚A·amu$^{1/2}$, of a proton moving in the hydrogen bond coordinate. Consequently, for a comparable excess vibrational energy, the proton-transfer seam is significantly more accessible and is therefore sampled more readily in the dynamics.

(2) Ring puckering suppression. The reviewer's concern about "fixing carbon coordinates" does not apply here: we did not constrain any nucleobase carbon atoms. The only fixed coordinate is the hydrogen atom used to cap the glycosidic valence (to represent the sugar linkage), which does not restrict the out-of-plane ring distortions underlying puckering. This is consistent with prior work, in which even more intrusive mechanical restrictions were introduced to emulate adenine's fused-ring constraint using

aminopyrimidine, without suppressing ring-puckering deactivation pathways. [9] A comment about this has been added to the text in page 6, line 122:

[...] where the isolated nucleobase decay route of ring puckering is not favoured due to a higher displacement along the mass-weighted coordinate, [8] not due to a constraint artifact, since fixing the saturating hydrogens does not prevent the ring-puckering deactivation pathways. [9]

Point 5

As the authors state at the end, the current model is not able to predict formation of pyrimidine dimers, which is an important lesion in DNA. Furthermore, I guess that excimer formation is neither properly described as the result of the geometrical constraints. Therefore, decay times are underestimated, is this right?

Response:

(1) CPD formation. Cyclobutane pyrimidine dimer formation is a low-yield photochemical channel: reported quantum yields for CPD formation are typically on the order of 1%, with strong dependence on sequence and structure. [10] With an ensemble of 100 trajectories, such a branching ratio is not statistically resolvable: even for a 1% yield, observing zero events remains plausible. Therefore, our trajectory set is appropriate to characterise dominant ultrafast deactivation pathways, but not to quantify rare photoproduct formation. The conclusion has been modified to underscore this fact in page 11, line 284:

Under the QM/MM framework, the explicit consideration of the DNA backbone in the MM region could modulate the conformational interplay of the nucleobase chromophores, [1] which, in turn, might affect the charge distribution during the dynamics and enable outcomes such as cyclobutane pyrimidine dimer (CPD) formation to be assessed. It should be taken into consideration, however, that given that the formation of CPDs is a relatively rare event, [2] running a statistically relevant number of trajectories would be required.

(2) Geometric constraints and decay time. Regarding the possibility that excimer formation is not accurately described due to geometric constraints, in our model, the only geometric restriction is applied to the capping hydrogen at the glycosidic attachment site, which we introduce to mimic the sugar linkage. Because this site is geometrically remote from the stacked π systems, it does not constrain the interbase motions (distance/slide/twist) that control excimer formation. Therefore, these geometric

constraints should not significantly impact decay times. This discussion is included next to the one on Point 4, regarding the effect of constraints, in page 6, line 125:

Likewise, these constraints should not affect which states are formed or the decay times, considering their distance to the π stacking sites that dominate interactions between bases.

References

[1] Lara Mart´ınez Fern´andez, Fabrizio Santoro, and Roberto Improta. Nucleic Acids as a Playground for the Computational Study of the Photophysics and Photochemistry of Multichromophore Assemblies. *Accounts of Chemical Research*, 55(15):2077–2087, August 2022.

[2] Takeshi Yanai, David P Tew, and Nicholas C Handy. A new hybrid exchange– correlation functional using the coulomb-attenuating method (cam-b3lyp). *Chemical physics letters*, 393(1-3):51–57, 2004.

[3] Felix Plasser, Adelia JA Aquino, William L Hase, and Hans Lischka. Uv absorption spectrum of alternating dna duplexes. analysis of excitonic and charge transfer interactions. *The Journal of Physical Chemistry A*, 116(46):11151–11160, 2012.

[4] Roberto Improta, Fabrizio Santoro, and Llu´ıs Blancafort. Quantum Mechanical Studies on the Photophysics and the Photochemistry of Nucleic Acids and Nucleobases. *Chemical Reviews*, 116(6):3540–3593, March 2016.

[5] Christian Wiebeler, Veniamin Borin, Adalberto Vasconcelos Sanchez de Araujo, Igor Schapiro, and Antonio Carlos Borin. Excitation energies of canonical nucleobases computed by multiconfigurational perturbation theories. *Photochemistry and photobiology*, 93(3):888–902, 2017.

[6] Miquel Huix-Rotllant, Johanna Brazard, Roberto Improta, Irene Burghardt, and Dimitra Markovitsi. Stabilization of Mixed Frenkel-Charge Transfer Excitons Extended Across Both Strands of Guanine–Cytosine DNA Duplexes. *The Journal of Physical Chemistry Letters*, 6(12):2247–2251, June 2015.

[7] Lara Martinez-Fernandez and Roberto Improta. Photoactivated proton coupled electron transfer in DNA: insights from quantum mechanical calculations. *Faraday Discussions*, 207(0):199–216, April 2018.

[8] Mario Barbatti, Ad´elia JA Aquino, Jaroslaw J Szymczak, Dana Nachtigallov´a, Pavel Hobza, and Hans Lischka. Relaxation mechanisms of uv-photoexcited dna and rna nucleobases. *Proceedings of the National Academy of Sciences*, 107(50):21453–21458, 2010.

[9] Mario Barbatti and Hans Lischka. Can the nonadiabatic photodynamics of aminopyrimidine be a model for the ultrafast deactivation of adenine? *The Journal of Physical Chemistry A*, 111(15):2852–2858, 2007.

[10] WJ Schreier, J Kubon, P Clivio, W Zinth, and P Gilch. Dna photodamage: Study of cyclobutane pyrimidine dimer formation in a locked thymine dinucleotide. *Journal of Spectroscopy*, 24(3-4):309–316, 2010.

jz-2026-00376u.R2

Name: Peer Review Information for "The Hidden Routes of DNA Photostability: Charge and Proton Transfer in Excited Cytosine-Guanine Tetramers"

Second Round of Reviewer Comments

Reviewer: 3

Comments to the Author

In the revision of the manuscript, the authors address all the points from the Reviewers. In most of them, a solution is provided. However, there is one point that I would like to emphasize again since it is not successfully addressed. My concern is that the preference proton transfer decay mechanism vs ring puckering decay in the isolated nucleobases might be associated to the deficiency of the level of theory. In Point 4 of the response letter,

the authors indicate that both proton-transfer and ring-puckering internal conversions are equally energetically accessible in their computations. Previous works on GC base pairs carried out with multireference methods, when analyzing the potential energy surfaces of the excited states upon the proton-transfer coordinate they find an energetic barrier to access the charge transfer state that needs to be populated to activate the proton-transfer decay mechanism. On the contrary, ring-puckering shows a barrierless or almost barrierless potential energy surface. Is this also the case for the methodology used in this work? I recommend to do such test calculation to have a direct comparison with another level of theory with higher accuracy and on the same molecular system (a single GC base pair). If the same trends are obtained, it would allow to clearly conclude that Wigner sampling, kinetic energy and dynamic effects are able to overcome the energy barrier to reach the CT state and therefore favor the proton-transfer mechanism. On the other hand, if in this test calculation, the CT is accessed without energy barrier or almost no barrier then the conclusion of the work would be biased by the used methodology and therefore the reliability would be questionable.

The previous findings obtained with high-accuracy methods point to ring-puckering as the one responsible for the photostability in nucleic acids. Since this manuscript change this knowledge, I consider important to clearly demonstrate that the findings are not a consequence of the level of theory. Upon clarifying this aspect, the work presented by the authors would be of great importance in the community establishing the current knowledge on DNA decay mechanisms.

Author's Response to Peer Review Comments:

Thank you for giving us the opportunity to submit a revised draft of the manuscript, and we also thank the reviewer for their additional comment. Please see attached a detailed response that addresses their point.

Response to reviewer report

The Hidden Routes of DNA Photostability: Charge and Proton

Transfer in Excited Cytosine-Guanine Tetramers

J. G. de Abrantes, J. M. Toldo, M. Barbatti, M. Sacchi

April 27, 2026

Thank you for giving us the opportunity to submit a revised draft of the manuscript "The Hidden Routes of DNA Photostability: Charge and Proton Transfer in Excited Cytosine-Guanine Tetramers". We thank Reviewer 3 for insisting on an important point and suggesting a reference (DOI: 10.1021/ct3006166) to support their comment, highlighted below in blue:

> *In the revision of the manuscript, the authors address all the points from the Reviewers. In most of them, a solution is provided. However, there is one point that I would like to emphasize again since it is not successfully addressed. My concern is that the preference proton transfer decay mechanism vs ring puckering decay in the isolated nucleobases might be associated to the deficiency of the level of theory. In Point 4 of the response letter, the authors indicate that both proton-transfer and ring-puckering internal conversions are equally energetically accessible in their computations. Previous works on GC base pairs carried out with multireference methods, when analyzing the potential energy surfaces of the excited states upon the proton-transfer coordinate they find an energetic barrier to access the charge transfer state that needs to be populated to activate the proton-transfer decay mechanism. On the contrary, ring-puckering shows a barrierless or almost barrierless potential energy surface. Is this also the case for the methodology used in this work? I recommend to do such test calculation to have a direct comparison with another level of theory with higher accuracy and on the same molecular system (a single GC base pair). If the same trends are obtained, it would allow to clearly conclude that Wigner sampling, kinetic energy and dynamic effects are able to overcome the energy barrier to reach the CT state and therefore favor the proton-transfer mechanism. On the other hand, if in this test calculation, the CT is accessed without energy barrier or almost no barrier then the conclusion of the work would be biased by the used methodology and therefore the reliability would be questionable.*

The previous findings obtained with high-accuracy methods point to ringpuckering as the one responsible for the photostability in nucleic acids. Since this manuscript change this knowledge, I consider important to clearly demonstrate that the findings are not a consequence of the level of theory. Upon clarifying this aspect, the work presented by the authors would be of great importance in the community establishing the current knowledge on DNA decay mechanisms.

Response:

We thank the reviewer for insisting on this important point. We agree that the relative energetics and ordering of locally excited (LE) and charge-transfer (CT) states are crucial for assessing the balance between proton-transfer and ring-puckering decay pathways.

To address this issue, we carried out additional test calculations on a GC dimer along the proton-transfer coordinate, comparing the TDDFT protocol used in this work with CASPT2. These tests show that the result depends strongly on the starting geometry. For pathways initiated at the S_0 Franck-Condon minimum, TDDFT places CT states lower than CASPT2 and yields a stronger CT contribution to S_1. In contrast, for pathways initiated from geometries drawn from the Wigner distribution, which are more representative of the initial conditions used in our dynamics, TDDFT no longer shows the same clear preference for CT, and LE states remain competitive in the S_1 region. Thus, the discrepancy between TDDFT and CASPT2 is significant, but it is not uniform across the region of configuration space sampled by the dynamics.

At the same time, we note that the relevance of this dimer benchmark to the present tetramer is necessarily limited. The tetramer has a much higher density of excited states and a larger hydrogen-bonding/stacking environment, both of which may substantially modify the balance between LE and CT characters. For this reason, the dimer calculations are useful to qualify the electronic-structure description, but they do not provide a definitive validation or invalidation of the tetramer mechanism.

We also agree with the reviewer that, at a higher level of theory, the competition between proton transfer and ring puckering could become more pronounced than in the present TDDFT-based dynamics. Our results should therefore not be interpreted as excluding such competition in DNA fragments more generally. Rather, they identify proton-transfer-driven decay as the dominant pathway within the present tetramer model and computational framework.

We have revised the manuscript accordingly to make the scope of our conclusions explicit and to clarify that the present work should be viewed as a TDDFT-based mechanistic picture for a DNA-motivated tetramer model. In our view, this remains valuable because it

establishes a tractable protocol for investigating competing deactivation channels in stacked hydrogen-bonded assemblies and provides a concrete basis for future higher-level benchmarks when such calculations become computationally feasible.

The new calculations are documented in Section 4 of the Supporting Information and have been added to the discussion in the first paragraph of page 7, highlighted below in red: Additional benchmark calculations on a GC dimer along the proton-transfer coordinate show that the relative ordering of locally excited (LE) and charge-transfer states depends on both the electronic-structure method and the starting geometry (see section 4 in the supporting information). In particular, TDDFT yields a stronger stabilisation of CT character than CASPT2 [1] for paths initiated at the Franck-Condon minimum, whereas for geometries drawn from the Wigner distribution, the LE states remain competitive in the S_1 region. These results indicate that the balance between LE and CT characters is method-dependent, but also sensitive to the part of configuration space sampled by the dynamics. Because the present tetramer has a much higher density of excited states and a larger hydrogen-bonding/stacking environment than the isolated GC dimer, these benchmark calculations do not provide a direct validation of the tetramer mechanism. They nevertheless show that the proton-transfer-driven decay reported here should be interpreted within the present TDDFT-based framework.

And in the concluding paragraph, page 12:
Finally, this work offers a framework for analysing such complex, multi-pathway dynamics rather than proposing a universal reassignment of DNA photostability mechanisms. It should be kept in mind that this mechanistic picture emerges for this tetramer model within the current computational framework.

References

[1] Vicenta Sauri, Jo˜ao P. Gobbo, Juan J. Serrano-P´erez, Marcus Lundberg, Pedro B. Coto, Luis Serrano-Andr´es, Antonio C. Borin, Roland Lindh, Manuela Merch´an, and Daniel Roca-Sanju´an. Proton/Hydrogen Transfer Mechanisms in the Guanine–Cytosine Base Pair: Photostability and Tautomerism. *Journal of Chemical Theory and Computation*, 9(1):481–496, January 2013.
